# Genomic insights and biotechnological potential of a saline soil fungus *Aspergillus tubingensis* FF14 for xanthophyll biosynthesis

**DOI:** 10.1128/spectrum.01179-26

**Published:** 2026-05-18

**Authors:** Weronika Śliżewska, Katarzyna Struszczyk-Świta, Joanna Oracz, Flavia Pinzari, Maurycy Daroch, Agata Czyżowska, Olga Marchut-Mikołajczyk

**Affiliations:** 1 Faculty of Biotechnology and Food Sciences, Institute of Molecular and Industrial Biotechnology, Lodz University of Technology, Lodz, Poland; 2 Faculty of Biotechnology and Food Sciences, Institute of Food Technology and Analysis, Lodz University of Technology, Lodz, Poland; 3 National Research Council of Italy (CNR), Institute for Biological Systems, Rome, Italy; 4 Natural History Museum, London, United Kingdom; 5 School of Environment and Energy, Peking University Shenzhen Graduate School 429362, Shenzhen, China; 6 Faculty of Biotechnology and Food Sciences, Institute of Fermentation Technology and Microbiology, Lodz University of Technology, Lodz, Poland; Connecticut Agricultural Experiment Station, New Haven, Connecticut, USA

**Keywords:** halophilic fungi, *Aspergillus tubingensis*, secondary metabolites, xanthophylls, genomics

## Abstract

Microbial pigment production is often a physiological response to environmental stress, including salinity, oxidative stress, and nutrient limitation. In this study, we investigated *Aspergillus tubingensis* FF14, a filamentous fungus isolated from naturally salinized coastal soil, periodically penetrated by seawater. This strain produced a distinctive orange intracellular pigment, which was identified via ultra-high-performance liquid chromatography coupled to diode array detection and electrospray ionization mass spectrometry as a mixture of three xanthophylls: violaxanthin, antheraxanthin, and neoxanthin. To date, this is the first documented case of xanthophyll biosynthesis in an *Aspergillus* species. Additionally, the antioxidant potential of the extracted pigment mixture was evaluated. Optimization experiments revealed that maximal pigment yield occurred at pH 3, 30°C, with 5% (vol/vol) sodium chloride, and 30% malt extract medium volume. Genome sequencing and annotation revealed biosynthetic gene clusters with domains characteristic of lycopene cyclase and phytoene synthase, indicating involvement in early steps of C40 carotenoid biosynthesis. Although biosynthetic gene clusters that are potentially linked to carotenoid biosynthesis were identified, the specific mechanisms responsible for xanthophyll biosynthesis in *A. tubingensis* FF14 remain to be elucidated. These findings provide new insights into fungal carotenoid metabolism and demonstrate the potential of *A. tubingensis* FF14 as a sustainable source of carotenoid pigments.

**IMPORTANCE:**

Microorganisms living in extreme environments frequently produce compounds with protective value against environmental stressors. Although these natural products have potential practical value, many remain undiscovered. We identified a halophilic fungus, *Aspergillus tubingensis* FF14, isolated from saline soil that produces bright orange pigments belonging to the xanthophyll family, a group of carotenoids with antioxidant potential. This is the first report on xanthophyll production in this genus. We showed that salt stress enhanced pigment production and optimized culture conditions to increase pigment yield. Genome analysis identified the genes that are most likely responsible for carotenoid biosynthesis, linking metabolite production with genetic potential. These findings expand current knowledge of fungal metabolites and demonstrate that extremophilic fungi may be promising and sustainable sources of valuable natural pigments.

## INTRODUCTION

Pigments are colorful substances that serve crucial functions essential for the survival of a whole range of organisms on Earth (1). Natural pigments vary in their biochemical roles and adaptive functions (2). Microorganisms are versatile producers of pigments and can synthesize a wide variety of them (3). For example, cyanobacteria contain pigments like chlorophyll concerns and phycobilin (4), while fungi can possess a wide range of melanins (5). These microbial pigments serve diverse biological functions, including metabolic regulation and protection from environmental stressors (6).

The adaptation mechanisms of extremophilic microorganisms to extreme environments trigger genetic and biochemical variations that lead to the synthesis of protective compounds, including pigments (7). Their unique characteristics can improve the production of pigments in highly acidic or alkaline environments, extreme temperatures, high pressures, and other unfavorable conditions (8). These microorganisms exhibit greater resistance to radiation, oxidative stress, ultraviolet light, and aggressive chemical processes, which are closely related to their ability to adapt to extreme environments (9).

Microorganisms adapted to saline environments—halophiles and halotolerants—are recognized as significant producers of melanins, carotenoids, and quinones (10, 11). Among these compounds, carotenoids play a crucial role by protecting cells against UV radiation and oxidative stress and contributing to membrane fluidity (12). Evidence suggests that carotenoids may offer protection against temperature and osmotic stress by enhancing the biosynthesis of pigments within this group (13).

Secondary metabolism is a key aspect of fungal physiology that supports adaptation to environmental stress together with promoting survival and reproduction (14). At the genomic level, fungal secondary metabolite biosynthesis is typically encoded by gene clusters, highlighting tight connections between metabolism, environmental adaptation, and pigment production (15). Pigments represent an important class of fungal secondary metabolites and are closely associated with stress tolerance (16). Carotenoids, in particular, are produced to serve as protective agents against environmental stress while also serving as precursors for biologically active apocarotenoids. Although carotenoids are not essential for growth, their biosynthesis is linked to ecological functions and the ability of fungi to withstand environmental challenges (17).

Carotenoids are synthesized by various halophilic bacteria, archaea, and microalgae (18–20); however, evidence regarding their production by halophilic fungi is still limited (21, 22). This is surprising considering fungi in different environments are documented to produce carotenoids, as indicated by several reports (23–25).

Production of yellow pigments by fungi of the *Aspergillus* group was reported as early as 1950, although many of these compounds were never chemically identified (26). A pigment named “asperxanthin,” likely belonging to acidic carotenoids (probably xanthophylls), was reported from *Aspergillus niger* but was never fully characterized (27, 28). Other authors suggested the production of pigments from the carotenoid group without more detailed identification (29, 30).

While carotenoid production has been described for mutant *Aspergillus* strains (31–34), there is a lack of recent studies demonstrating carotenoid biosynthesis in wild-type *Aspergillus* environmental isolates, for which ecological adaptation mechanisms may be responsible for pigment biosynthesis. To date, carotenoid production has not been reported for *A. tubingensis* strains.

The objective of this research was to investigate the potential of the strain *Aspergillus tubingensis* FF14 for carotenoid pigment production. The study aimed to evaluate pigment biosynthesis, identify its chemical structure and potential biological function, and improve the conditions for its production. Furthermore, a bioinformatic analysis was conducted based on the whole-genome sequencing of the isolate *A. tubingensis* FF14 to provide insights into the genetic basis of pigment biosynthesis and related metabolic pathways. Comparative analysis of *A. tubingensis* genomes was conducted to determine potential genes and biosynthetic gene clusters (BGCs) responsible for the biosynthesis of carotenoid pigments in *A. tubingensis* FF14.

## RESULTS

### Characteristics and identification of the pigment-producing strain FF14

Strain FF14 was isolated as part of a survey investigating microbial biodiversity and physiological traits of halophilic and halotolerant fungi obtained from saline soils with various pedoclimatic characteristics (35). Our preliminary assessment revealed a strong pigment production capacity for this strain.

**Fig 1:**
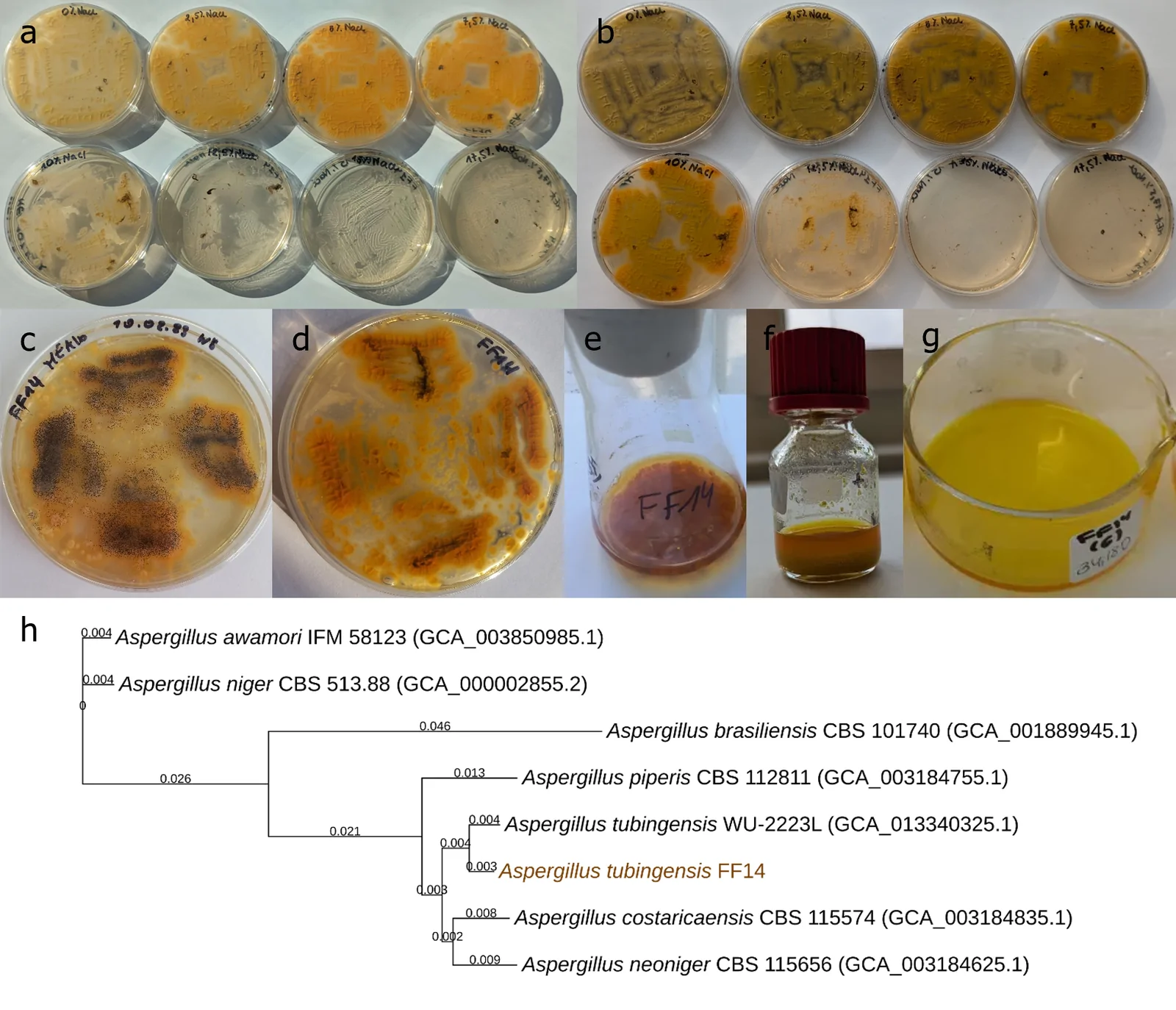
Pigment production by *A. tubingensis* FF14 and its phylogenetic tree. (**a and b**) Results of halotolerance test – growth on MEA supplemented with different NaCl concentrations after 4 days (**a**) and 7 days (**b**). Upper row represents, from left: no NaCl addition to media, MEA with 2.5% NaCl, followed by 5 and 7.5%. On the lower row, from left: MEA supplemented with 10%, 12.5%, 15%, and 17.5% NaCl; (**c and d**) fungal culture of *A. tubingensis* FF14 on MEA + 10% NaCl after 10 days; (**e**) growth of pigment-producing fungi in liquid ME medium; (**f**) a mixture of the fungal biomass and the solvent (acetone/methanol [7:3, vol/vol]) during pigment extraction; (**g**) extracted pigment after biomass separation; (**h**) phylogenetic tree of *A. tubingensis* FF14 and closely related *Aspergillus* strains based on whole-genome sequences. The tree was generated using RealPhy based on maximum-likelihood algorithm with whole-genome sequence alignments and visualized using iTOL v.6. Values on the nodes indicate branch lengths.

While growing on malt extract agar (MEA) plates, *A. tubingensis* FF14 appeared as orange-colored mycelium with black conidia characteristic of *Aspergillus* section *Nigri* strains. During the halotolerance test assay carried out in preliminary research (35), mycelial pigmentation was observed at different salt concentrations, with the pigment being visible only under moderate salinity conditions (2.5%–12.5% [wt/vol] NaCl) and no or minimal amount of pigment being formed at all on MEA without added salt or at high salt concentrations, as presented in Fig. 1a and b. It was also noted that pigment was only intracellular, as pigment was not detected in cell-free supernatant. The strain’s growth, pigment production, and pigment during extraction process are shown in Fig. 1c through g. The average wet biomass production was 29.25 ± 5.36 g/L in liquid medium. The concentration of extracted intracellular pigment was 20.35 ± 2.67 mg/g of wet biomass, and 0.61 ± 0.18 g/L of culture.

Strain FF14 was initially identified as *Aspergillus neoniger,* mostly based on internal transcribed spacer (ITS) and translation elongation factor 1-alpha (TEF1-α) nucleotide sequences. However, further molecular identification, based on whole-genome sequences conducted as part of this study, demonstrated a close relation of strain FF14 to *Aspergillus tubingensis*, leading to the reidentification of the strain. The results of whole-genome matching are presented in Table S1, and the phylogenetic tree constructed from whole-genome sequence data is shown in Fig. 1h.

### Pigment analysis

**Fig 2:**
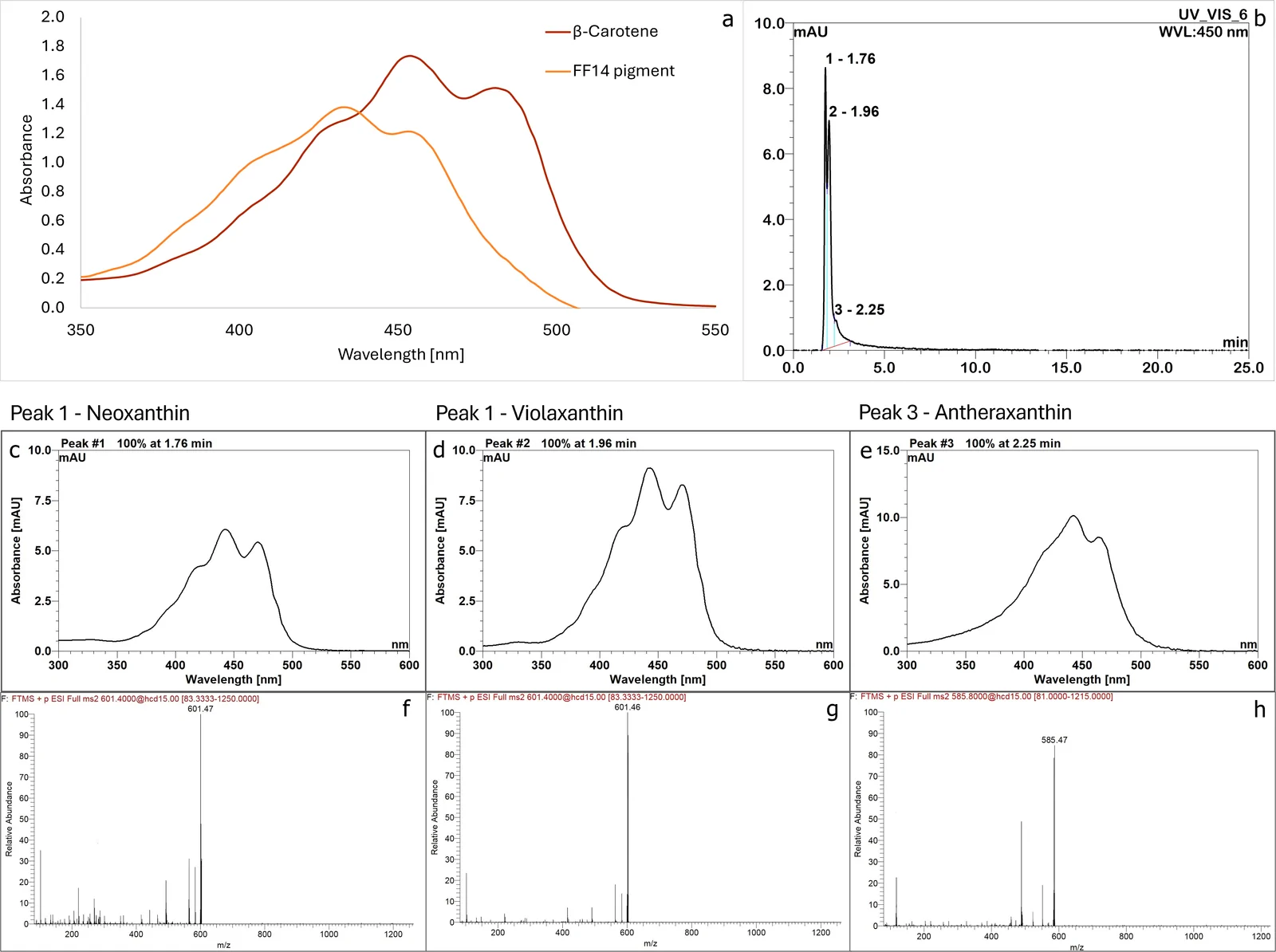
Characterization of obtained *A. tubingensis* FF14 pigment. (**a**) UV-Vis spectra of the obtained pigment from *A. tubingensis* FF14 compared to β-carotene standard; (**b**) UHPLC-DAD spectra of pigment of *A. tubingensis* FF14; UV-Vis (**c–e**) and mass spectra (**f–h**) of obtained compounds by UHPLC-DAD-ESI-MS/MS: neoxanthin (**c and f**), violaxanthin (**d and g**), and antheraxanthin (**e and h**).

The UV-Vis spectrum of the obtained pigment and β-carotene standard was analyzed from 350 to 550 nm, as presented in Fig. 2a. The maximum absorbance for the β-carotene standard was determined to be at 454 nm, with secondary peaks at 481 nm and about 420 nm–430 nm, while for *A. tubingensis* FF14 pigment, the main peak was at 433 nm and secondary peaks at 453 nm and about 400 nm–410 nm. Those UV-Vis absorption peaks seem to correspond to typical spectra of carotenoids (λ_max_ between 400 and 500 nm; β-carotene maximal peaks in the range of 450 nm–480 nm) and yellow xanthophylls (λ_max_ between 420 and 480 nm) (36, 37).

The characterization of the colored compounds isolated from *A. tubingensis* FF14 was later carried out by ultra-high-performance liquid chromatography coupled to diode array detection and electrospray ionization mass spectrometry (UHPLC-DAD-ESI-MS) analysis. Compounds were identified based on retention times, UV-Vis spectra, full mass spectra, and fragmentation spectra (Table 1; Fig. 2b through h), which were compared with literature data (38–41). To further strengthen identification, results were compared with extracts obtained from yellow bell pepper and spinach, which were analyzed under identical chromatographic conditions (Fig. S1 to S3). Yellow bell pepper and spinach were selected as well-established and widely accepted natural sources of violaxanthin, antheraxanthin, and neoxanthin (42, 43).

**TABLE 1:** Compounds identified using the UHPLC-DAD-ESI-MS/MS method in the FF14 sample

Peak no.	t_R_ (min)	Identified compounds	UV absorbance (λmax, nm)	[M + H]^+^ (*m/z*)	MS^2^ fragments (*m/z*)	References
1	1.76	Neoxanthin isomer	439.9, 468.8	601.47	583, 565, 491, 221	(39, 44–46)
2	1.96	Violaxanthin isomer	443.0, 469.9	601.46	583, 565, 491, 221	(39, 40, 44–46)
3	2.25	Antheraxanthin isomer	438.2, 468.9	585.47	568, 550, 533, 493	(40, 44, 47)

As a result, three compounds were determined and identified as xanthophylls. Neoxanthin isomer (peak 1) was identified based on the protonated molecule ([M + H]^+^) at m/z 601.47 and its characteristic UV-visible spectrum. The molecular mass of the neoxanthin isomer was also confirmed by fragments indicating consecutive losses of two hydroxyl groups from the protonated molecule (m/z 583 and 565), the loss of toluene followed by the loss of water (m/z 491), and cleavage of the polyene chain (m/z 221). On the same basis, violaxanthin (peak 2) was identified with [M + H]^+^ at m/z 601.46 and corresponding fragments at m/z 583, 565, 491, and 221. Antheraxanthin (peak 3) with [M + H]^+^ at m/z 585.47 also exhibited fragments due to losses of hydroxyl groups (m/z 568, 550, 533) and an additional loss of toluene (m/z 493). For suspected xanthophylls, UV-Vis absorption peaks typically appear at wavelengths around 415, 440, and 468 nm (48, 49). Neoxanthin, antheraxanthin, and violaxanthin are well-documented to elute in close proximity to one another across various chromatographic conditions, including standard high-performance liquid chromatography (HPLC) methods, typically within a timeframe of 1 to 3 min (44–47, 50, 51). Consequently, accurate identification of these peaks necessitated a detailed analysis of UV-Vis spectra and MS/MS fragmentation patterns, which were compared against multiple literature sources (listed in Table 1). The shift of absorption maxima of extracted pigments can vary depending on the solvent used, the other compounds present in the sample, and the type of spectrophotometer used for measurements (52).

### Genome assembly

The genome of the fungal strain FF14 isolated from saline soil in Italy was sequenced using Illumina-type technology with a coverage of 192×. The obtained genomic data were assembled at the contig level, yielding a genome size of 35.6 Mb comprising 129 contigs. Statistics of genome assembly and general annotation features are presented in Table 2.

**TABLE 2:** Genome characteristics of *A. tubingensis* FF14

Feature	Value
Accession number	GCA_050924195.1
Coverage (×)	192
# Contigs	129
Largest contig	1,405,748
Total length (Mb)	35.6
N50	467,307
N90	149,605
L50	25
L90	77
GC (%)	48.99
BUSCO (%)	99.0
Protein-coding genes	10,010
Number of tRNA genes	293

A total of 3,512 groups of genes were obtained, indicating good genome completeness. The annotation performed with AUGUSTUS predicted 10,578 coding genes, among which 85.14% (8,523 matching genes) were assigned to Cluster of Orthologous Groups (COG) functions, 63.15% (6,321 matching genes) to gene ontology (GO) categories, 39.97% (4,001 matching genes) to Kyoto Encyclopedia of Genes and Genomes (KEGG), and 5.21% (522 matching genes) to carbohydrate-active enzymes database (CAZymes).

### Genome annotations

**Fig 3:**
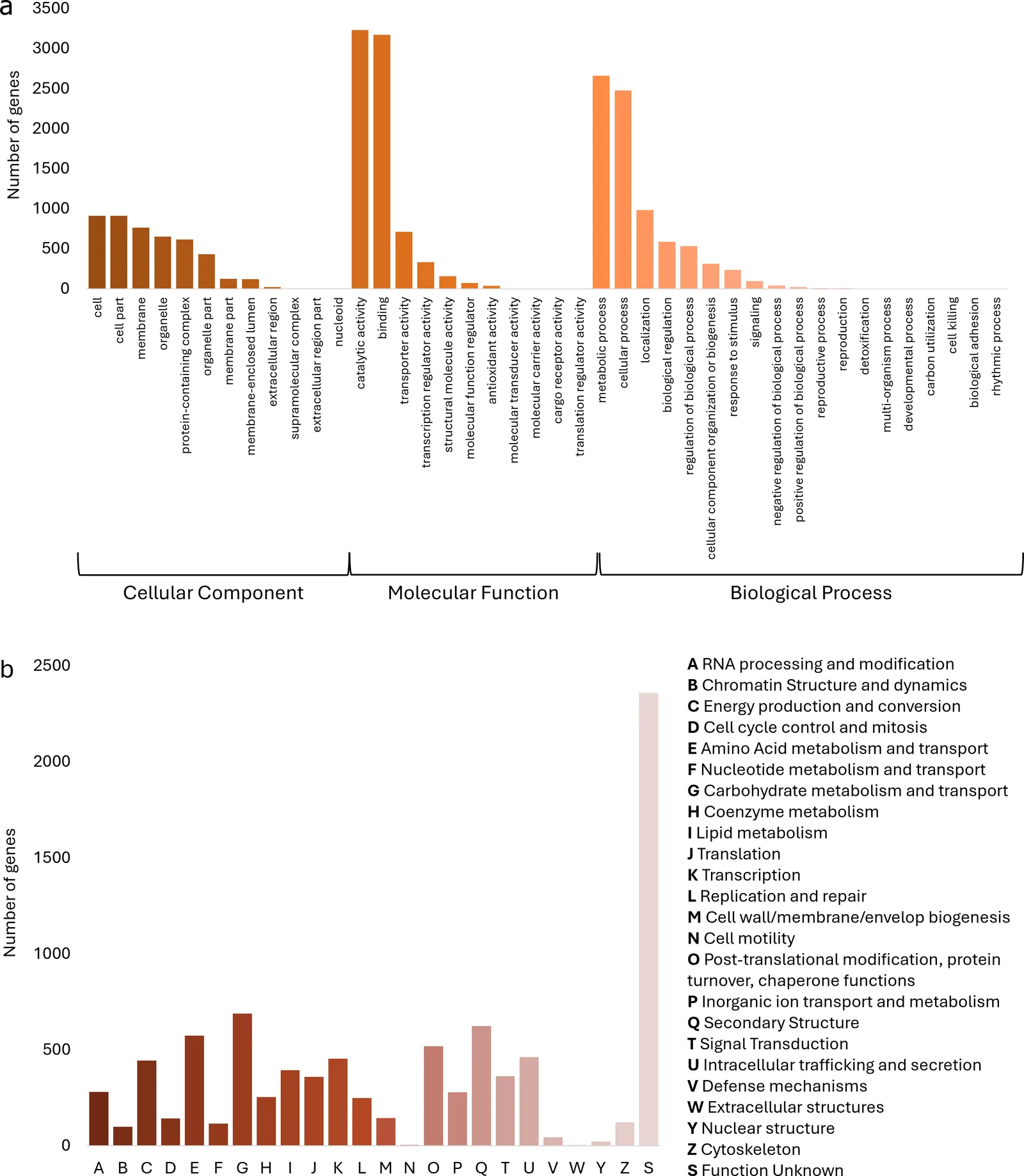
Annotation and functional classification of *A. tubingensis* FF14 – (**a**) GO; (**b**) histogram of COG.

Functional annotation based on GO and COG classifications is presented in Fig. 3.

The GO classification (Fig. 3a) reveals a dominant presence of genes involved in catalytic activity and binding. Among catalytic activities, hydrolase (1,083 genes) and oxidoreductase (989 genes) activities were the most abundant. Functions related to oxidative stress response and transport were also identified, along with genes associated with regulation, localization, and environmental response.

From the COG analysis (Fig. 3b), one of the most notable features is the high number of genes classified under carbohydrate metabolism and transport (category G). A high abundance of amino acid metabolism and transport (category E) was also detected. Additionally, a considerable proportion of genes were assigned to the “Function Unknown” category.

Functional classification of the annotated genes obtained from the Kyoto Encyclopedia of Genes and Genomes is shown in Fig. S4. A total of 4,001 genes (40.85%) were assigned to the Metabolism category, including genes involved in carbohydrate metabolism (276 genes), amino acid metabolism (329 genes), xenobiotic degradation (69 genes), and secondary metabolite biosynthesis (308 genes). The Environmental Information Processing category comprised 489 genes, predominantly associated with signal transduction pathways (423 genes).

**Fig 4:**
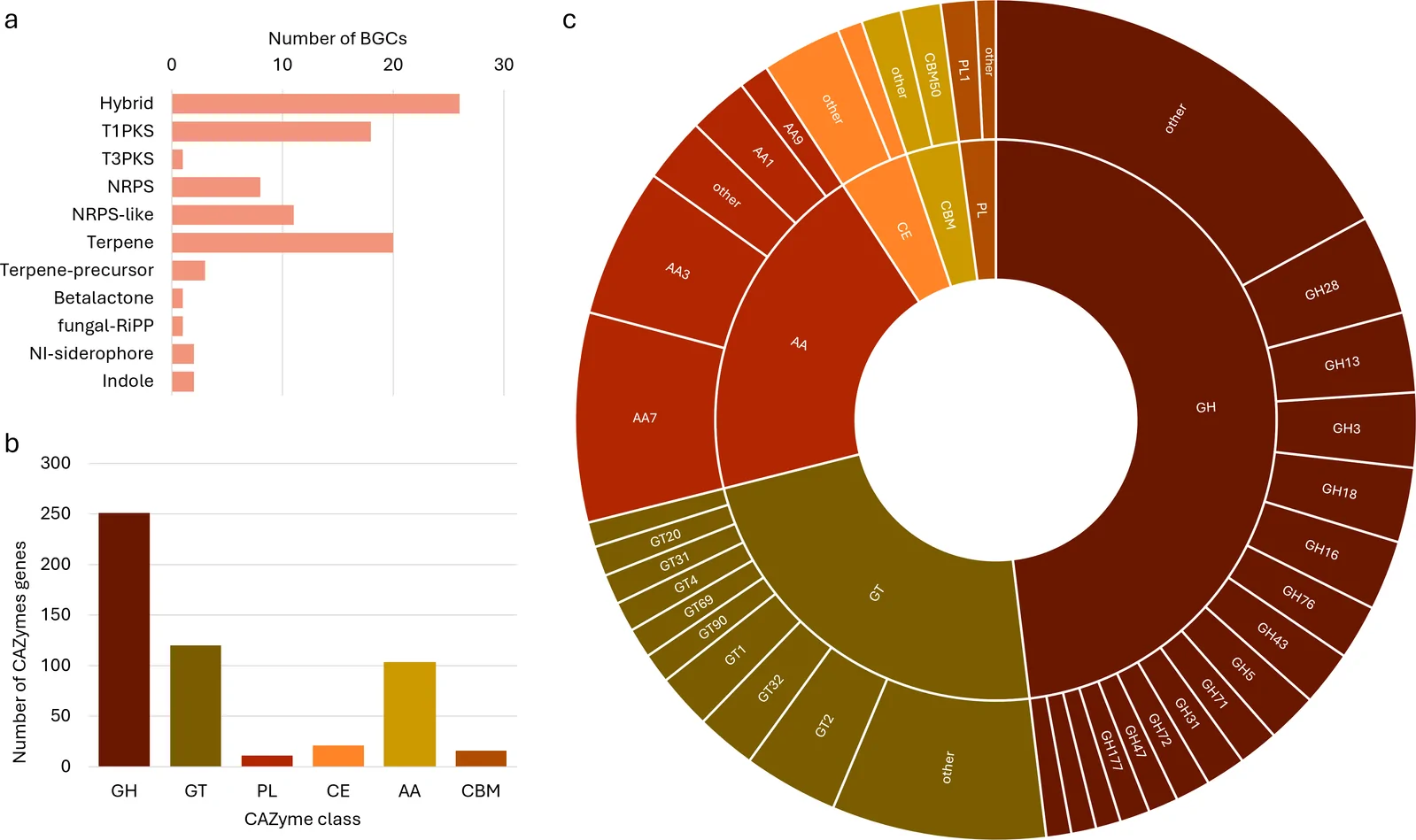
Summary of (**a**) antiSMASH results and (**b and c**) predicted CAZymes in *A. tubingensis* FF14 obtained from dbCAN. CAZyme classes: GH, glycoside hydrolases; GT, glycosyl transferases; AA, auxiliary activities; CE, carbohydrate esterases; CBM, carbohydrate-binding modules; PL, polysaccharide lyases.

AntiSMASH analysis revealed the presence of 93 BGCs, of which the majority corresponded to hybrid, non-ribosomal peptide synthetase (NRPS), and terpene clusters (Fig. 4a; Table S2). The number and types of BGCs were also compared with different *A. tubingensis* strains revealing consistent and comparable results (Fig. S5). Among the obtained clusters, two in particular (contig_43 – Region 1 and contig_70 – Region 1) attracted attention due to the presence of terpene synthase genes that may play a role in the biosynthesis of carotenoid precursors. A thorough analysis of those clusters is presented in the following section.

The dbCAN-based annotation of carbohydrate-active enzymes identified 522 putative CAZyme hits, as determined by at least two different tools (Fig. 4b and c). Glycoside hydrolases (GHs) were found to be the most abundant group. Among subclasses, AA7, AA3, GH28, and GT2 were the most dominant.

### Genomic analysis of carotenoid biosynthesis

A more in-depth analysis of functional annotations of *A. tubingensis* FF14 genome revealed the presence of genes involved in the biosynthesis of carotenoids and apocarotenoids. Predicted genes encoding phytoene desaturase (K15745), 5-cis-phytoene synthase/lycopene β-cyclase (K17841), (+)-abscisic acid 8′-hydroxylase (K09843), and β-apo-4′-carotenal oxygenase (K17819) were identified, suggesting the presence of a core pathway for the synthesis and oxidative cleavage of carotenoids (Fig. S6). To confirm the pathway assignment, InterPro and UniProt analyses were performed on the proteins of interest, in addition to the genes identified through KEGG mapping. This analysis revealed a broader range of genes potentially involved in carotenoid biosynthesis (Table 3). The contig_38.g6494 gene revealed a carotenoid oxygenase (IPR004294), a family of enzymes responsible for the oxidative cleavage of numerous carotenoids including β-carotene, lycopene, and zeaxanthin (53). Further UniProt analysis identified this gene as 9-*cis*-epoxycarotenoid dioxygenase, which is a key enzyme involved in the biosynthesis of abscisic acid (ABA) via cleavage of 9-*cis*-epoxycarotenoids (54). Genes predicted to encode phytoene desaturase (contig_15.g3334, contig_43.g6994, and contig_70.g8713) containing carotenoid/retinoid oxidoreductase domains (IPR014105), which is consistent with the desaturation activity of this enzyme (55). Contig_11.g2753 was annotated as squalene/phytoene synthase (IPR002060), an enzyme involved in the carotenoid pathway (56, 57). Contig_70.g8712, associated with 5‑cis‑phytoene synthase/lycopene β‑cyclase (K17841), encodes a protein containing a lycopene cyclase domain (IPR017825), a squalene/phytoene synthase domain (IPR002060), and a trans‑isoprenyl diphosphate synthase domain (IPR033904), indicating a role in carotenoid precursor biosynthesis (58). These enzymes’ multifunctionality serves a role in initiating carotenoid biosynthesis and catalyzing cyclization reactions (59).

**TABLE 3:** List of genes involved in the carotenoid metabolic pathways—production of carotenoids and their further biosynthesis into secondary metabolites^*a*^

Genes list	Length		InterPro		UniProt
	NT	AA	Entry ID	Gene description	Protein name
Contig_38.g6494	1,816	597	IPR004294	Carotenoid oxygenase	9-*Cis*-epoxycarotenoid dioxygenase
Contig_15.g3334	1,646	541	IPR014105	Carotenoid/retinoid oxidoreductase	Phytoene desaturase (3,4-didehydrolycopene-forming)
Contig_43.g6994	1,321	434			
Contig_70.g8713	1,515	498			
Contig_11.g2753	1,199	394	IPR002060	Squalene/phytoene synthase	Squalene/phytoene synthase
Contig_70.g8712	1,467	489	IPR017825	Lycopene cyclase domain	Bifunctional lycopene cyclase/phytoene synthase
			IPR002060	Squalene/phytoene synthase	
			IPR033904	Trans-isoprenyl diphosphate synthases, head-to-head	
Contig_2.g576	1,564	514	IPR012394	Aldehyde dehydrogenase NAD(P)-dependent	Aldehyde dehydrogenase

^*a*^ NT, nucleotides; AA, amino acids.

### Comparative analysis of *A. tubingensis* genomes

A total of 22 genomes belonging to *A. tubingensis* were analyzed to determine potential genes and biosynthetic gene clusters responsible for xanthophyll production in *A. tubingensis* FF14. Genomes were obtained from the NCBI database and were listed in Table S3.

**Fig 5:**
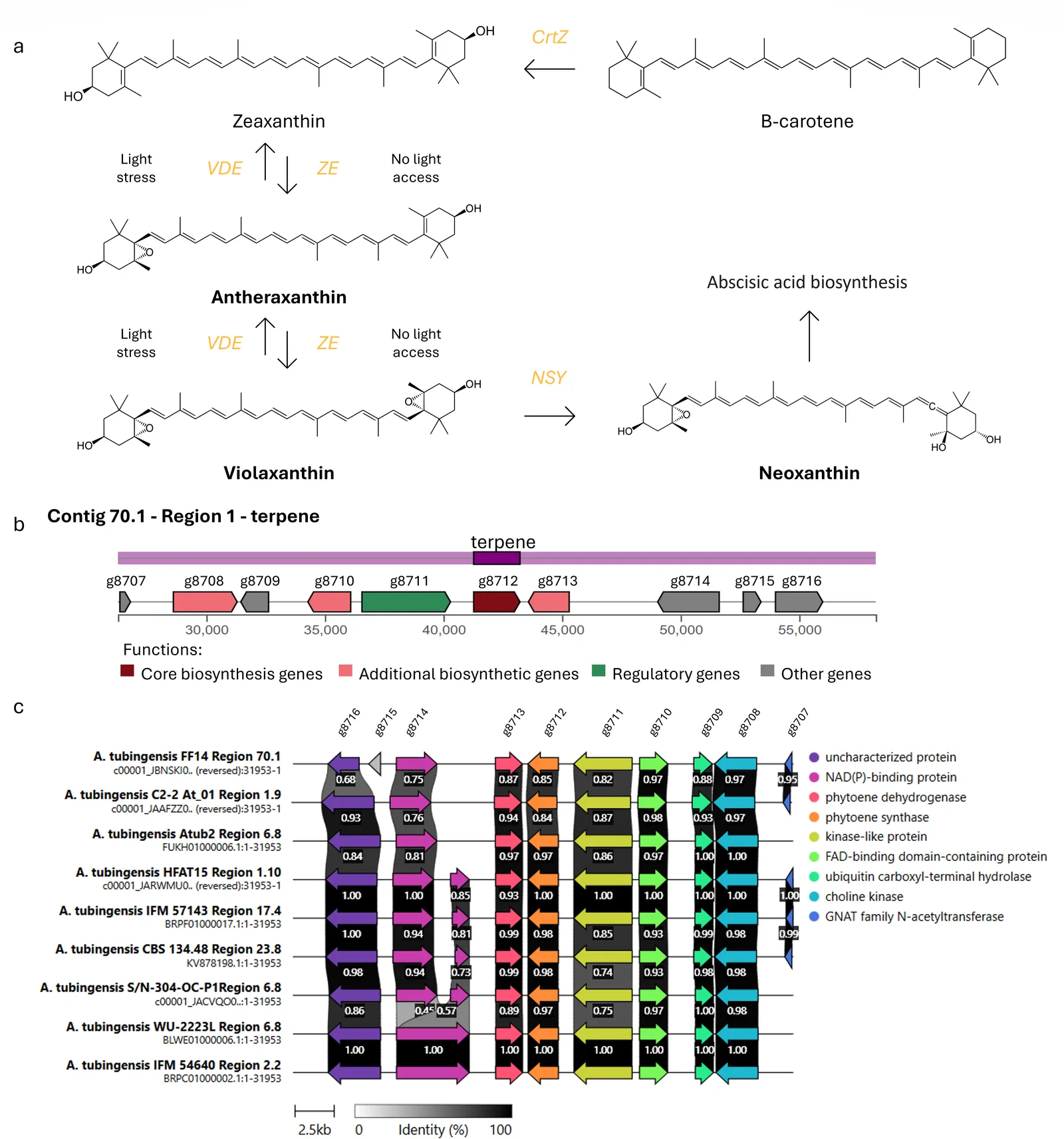
Analysis of carotenoid- and xanthophyll-related pathways and gene clusters. (**a**) The pathway for xanthophylls biosynthesis in plants and microorganisms. Xanthophyll cycle (zeaxanthin, antheraxanthin, violaxanthin) is produced from β-carotene and is part of the abscisic acid biosynthesis pathway. Enzymes involved in this branch of the carotenoid pathway: β-carotene hydroxylase (CrtZ), violaxanthin de-epoxidase (VDE), zeaxanthin epoxidase (ZE), and neoxanthin synthase (NSY). Compounds obtained in this study are marked in bold; (**b**) Predicted terpene cluster in contig 70 of *A. tubingensis* FF14; (**c**) Alignment and organization of related gene clusters to cluster (contig_70.1) from *A. tubingensis* FF14. Above graph, gene labels for *A. tubingensis* FF14 are shown.

Cluster contig_70 – Region 1 has been predicted to be associated with the biosynthesis of carotenoids (Fig. 5b). A comparison of the cluster of contig_70 with closely related *A. tubingensis* strains revealed high similarity to other clusters (Fig. 5c). The primary biosynthetic gene showed high similarity across all analyzed strains. Contig_70.g8712 encodes a protein with a lycopene cyclase domain (residues 53–144) and a squalene/phytoene synthase domain (residues 200–476). The lycopene cyclase domain mediates the cyclization of linear carotenoids, while the squalene/phytoene synthase catalyzes the condensation of two molecules of geranylgeranyl diphosphate (GGPP) to form phytoene, the first committed precursor of C40 carotenoid biosynthesis. The functional potential of this gene encompasses the formation of intermediates such as squalene, diapophytoene, presqualene diphosphate (PSPP), and phytoene, which represent key branching points for the synthesis of downstream xanthophylls. Additionally, contig_70.g8707, which was present only in three other strains, encoded a GNAT family N-acetyltransferase. However, contig_70.g8715 did not show as low as 0.3 sequence identity alignment with any of the genes of other strains.

Orthologous genes were identified using OrthoFinder, which revealed 11,015 orthogroups, with 8,409 of them present in all species. Most of the tested genes were classified into orthogroups, with only 0.2% of unique genes within tested *A. tubingensis* strains. In Fig. S7, the heatmap showing the pairwise overlap of orthogroups among the *A. tubingensis* species indicated that strain FF14 shared the most orthogroups with strains Atub2 (9,617 orthogroups) and CBS 134.48 (9,592 orthogroups). Further analysis of the obtained results for *A. tubingensis* FF14 revealed that no species-specific orthogroups were identified for this strain. However, 18 genes of the FF14 strain were not assigned to any orthogroup. Most of these genes were found to encode transcription factors and regulatory proteins, while our genes were similar to uncharacterized proteins (Table S4). Among unassigned genes, three were part of previously identified biosynthetic gene clusters. Contig_4.1.g1005 was characterized as “other” function gene with NRPS-like, T1PKS cluster in contig_4 – Region 1, which showed high similarity to known cholin-related clusters' which keep information about choline clusters. A UniProt Blast identified this gene to encode a cyanovirin-N domain-containing protein. Contig_64.g8436 served a biosynthetic-additional function in the terpene-precursor cluster located in contig_64 – Region 1. Due to its identification as a C2H2-type domain-containing protein, it likely served as a transcription factor. Special attention should be given to contig_70.g8715. This gene was identified as part of the contig_70 – Region 1 cluster, from which other genes (contig_70.g8713 and contig_70.g8712) were already identified as potentially related to carotenoid metabolic pathways. Furthermore, this gene did not show identity alignment with any other gene from corresponding *A. tubingensis* clusters. Initially, this gene was identified as an uncharacterized protein. However, it also showed similarity to the Celp0028 effector-like protein.

### Determination of the best conditions for xanthophylls production

In this study, the Taguchi method was employed to identify the factors that improved xanthophyll production, specifically pH, temperature, NaCl concentration, and aeration based on volume (Table S5). Each of the four factors was tested at four different levels, resulting in 16 unique experiments (Table S6).

The results indicated that the best conditions for pigment production under the considered conditions were A1B3C2D3, corresponding to a pH of 3, a temperature of 30°C, a 5% vol/vol NaCl addition, and a 30% volume of the ME medium in the flask. The analysis of variance (ANOVA) revealed that each factor had a *P*-value of less than 0.05, confirming a significant relationship between the factors and the results. The contribution ratios for each factor indicated that temperature and NaCl concentration had the most substantial impact on xanthophyll production, with contributions of 35.72% and 29.19%, respectively. The pH of the medium contributed the least, at 8.05%, while the volume of the medium was also not very influential, contributing 10.71%. The contribution of the error was 16.33%.

Analyzing the main effect plots of each factor on the signal-to-noise (S/N) ratio (Fig. S8), it was determined that pH did not significantly influence pigment production, as both pH levels of 3 and 7 produced similar responses. Neither excessively low nor excessively high pH levels improved or reduced pigment production. In contrast, both temperature and NaCl concentration yielded the highest S/N ratios under optimal or slightly stressful growth conditions for *A. tubingensis* FF14, with extreme conditions having a notably negative impact on pigment production.

The cultivation and pigment extraction were performed under the designed best conditions determined by the Taguchi method. As a result of this optimization experiment, the efficiency of pigment extraction from the mycelium improved to 39.74 ± 7.92 mg/g of wet biomass, and to 2.00 ± 0.35 g of pigment per liter of culture. Consequently, under the improved conditions for the growth and pigment production of *A. tubingensis* FF14, pigment production yield rose three times for milligrams of pigment per milliliter of culture compared to the previous growth conditions used.

### Antioxidant properties

**Fig 6:**
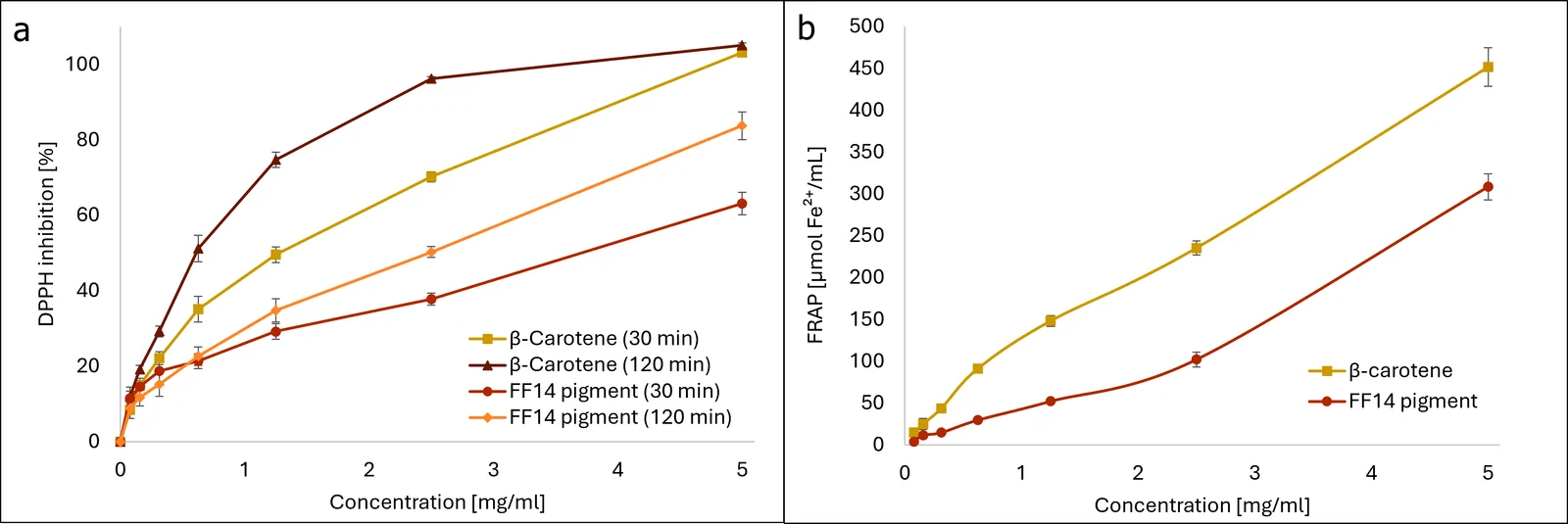
Antioxidant activity assays for xanthophyll pigment mixture from *A. tubingensis* FF14 and β-carotene standard. (**a**) scavenging of DPPH radicals, and (**b**) scavenging of FRAP radicals. Values are shown as the mean ± standard deviation from three independent experiments.

The antioxidant ability of the pigment from the FF14 strain was evaluated using the 2,2-diphenyl-1-picrylhydrazyl (DPPH) and ferric reducing antioxidant power (FRAP) assays for potential industrial uses, such as food additives (60). The results (Fig. 6a) revealed that the tested mixture of xanthophylls significantly reduced DPPH across all concentrations (5.00 to 0.078125 mg/mL), achieving the highest DPPH radical scavenging activity of 63.17% after 30 min and 83.77% after 120 min for the 5 mg/mL pigment solution. Additionally, it was observed that DPPH inhibition increased considerably over time. The inhibitory concentration (IC_50_) of the mixture required to scavenge 50% of free radicals for the DPPH assay was determined, showing that the FF14 pigment had an IC_50_ of 3.71 ± 0.07 mg/mL after 30 min and 2.47 ± 0.12 mg/mL after 120 min, compared to β-carotene with an IC_50_ of 1.28 ± 0.10 and 0.62 ± 0.12 mg/mL after 30 and 120 min, respectively. Those results are consistent with the antioxidant capacity evaluated using the FRAP assay (Fig. 6b). At all tested concentrations, β-carotene showed significantly higher FRAP values compared to the FF14 pigment extract. At the highest tested concentration of 5 mg/mL, β-carotene obtained a maximum FRAP value of 451.52 ± 23.02 µmol Fe²^+^/mL, while the FF14 pigment exhibited a lower but substantial activity of 308.57 ± 15.47 µmol Fe²^+^/mL.

## DISCUSSION

This study describes the first documented case of xanthophyll production by *Aspergillus tubingensis* FF14. It expands current knowledge of fungal carotenoid diversity and highlights a previously unreported metabolic capability within the genus *Aspergillus*. Although some fungal carotenoids have been identified and their biosynthetic pathways established, the majority of fungal carotenoids remain unknown, with pathways, enzymes involved in their production, and even chemical structures still undescribed (25). To date, there is no evidence that filamentous fungi strains naturally produce neoxanthin, antheraxanthin, or violaxanthin (17), pigments that are primarily associated with plants, algae, and photosynthetic microorganisms.

Violaxanthin and antheraxanthin, whose presence in the pigment mixture from *A. tubingensis* FF14 is suggested by the UHPLC-DAD-ESI-MS/MS results, together with zeaxanthin (not detected as part of this mixture), are pigments mostly associated with the xanthophyll cycle (61). They are primarily produced by plants, algae, and photosynthetic bacteria, serving them as photoprotection (62). Neoxanthin is a xanthophyll synthesized from violaxanthin, and it has a role in photooxidative protection. It is an intermediate in the production of ABA, a phytohormone important for plants to help them deal with environmental stresses, including salinity stress (63). Microbial violaxanthin, antheraxanthin, or neoxanthin were predominantly found in microalgae and bacteria. Violaxanthin has been produced by marine microalgae, including *Eustigmatos* cf. *polyphem* (64), *Nannochloropsis oceanica* (65), while *Chlamydomonas* sp. showed the ability to produce neoxanthin, violaxanthin, and antheraxanthin (66, 67). Among microalgae, isolated from saltern ponds, *Dunaliella viridis* produced all three detected xanthophylls under high salinity conditions (68). In prokaryotes, neoxanthin production has been described in the marine bacterium *Bacillus marisflavi* (69), while among archaea, the halophilic *Haloferax mediterranei* was reported to produce antheraxanthin under optimal growth conditions (70). These reports indicate that microbial production of pigments associated with the xanthophyll cycle is mostly associated with phototrophic or halophilic organisms, highlighting the rarity of such pigments in fungi.

Production of violaxanthin and antheraxanthin is usually related to the xanthophyll cycle of zeaxanthin, which is produced from β-carotene (Fig. 5a). Zeaxanthin could be produced as it is in plants with β-cryptoxanthin as an intermediate using β-carotene hydroxylases (CrtB and CrtZ) and zeaxanthin epoxidase (ZEP, EC 1.14.15.21) genes, or directly catalyzed by CrtZ as in the bacterial pathway (71–73). Violaxanthin is synthesized from zeaxanthin with antheraxanthin as an intermediate by zeaxanthin epoxidase. Under strong light conditions in this convertible reaction, violaxanthin can be converted back to zeaxanthin via antheraxanthin by violaxanthin de-epoxidase (VDE, EC 1.23.5.1) (74). Neoxanthin is further synthesized from violaxanthin by neoxanthin synthase (NSY) as part of the biosynthesis pathway leading to the production of abscisic acid (72). Initial genomic studies focused on key enzymes responsible for the production of xanthophyll cycle compounds, but genomic analysis was expanded to the entire carotenoid pathway, recognizing the interconnected nature of carotenoid biosynthesis. Metabolic profiling of these compounds in *A. tubingensis* FF14 suggests the existence of alternative or highly divergent enzyme homologs that cannot be easily detected by standard sequence similarity or domain-based annotation tools. This disconnect between secondary metabolite production and gene annotation highlights the complexity of carotenoid metabolism in fungi and suggests that unexplored metabolic pathways may be present in this strain. Research has not yet shown the production of pigments from the xanthophyll group in fungi belonging to the *Aspergillus* genus.

The biosynthesis pathway of carotenoids in fungi, leading to β-carotene and torulene, occurs in three main steps. First, two molecules of geranylgeranyl pyrophosphate are condensed to phytoene. Next, a single enzyme catalyzes four desaturation steps. Finally, two enzymes cyclize the compound into a β-ionone group. This process of fungal carotenoid biosynthesis is typically regulated by two conserved enzymes: a single phytoene desaturase (AL-1, crtI, or CarB) and a bifunctional phytoene synthase/lycopene cyclase (AL-2, crtYB, or CarRA) (25, 75–77). Our genome analysis demonstrates that *A. tubingensis* FF14 possesses both core genes: CrtI-type phytoene desaturases (contig_15.g3334; contig_43.g6994; contig_70.g8713) and bifunctional CrtYB/CarRA-type synthase/cyclases (contig_70.g8712), which is consistent with the conserved fungal pathway reported by Stra et al. (78).

The presence of these genes indicates *A. tubingensis* FF14’s ability to produce carotenoid-derived compounds and may underlie the strain’s ability to synthesize a diverse set of carotenoid compounds. Additionally, some of the genes, for example, carotenoid oxygenases, may not be involved in the production of carotenoids but rather in their cleavage and further biosynthesis of apocarotenoids such as abscisic acid. However, despite the detection of multiple genes related to the core carotenoid pathway, no direct genetic evidence was found for genes typically involved in the biosynthesis of specific downstream xanthophylls such as violaxanthin, antheraxanthin, or neoxanthin, whose production was confirmed in this study.

Functional annotation cluster contig_70 – Region 1 suggests it may play a role in carotenoid biosynthesis, specifically in the formation of C40 carotenoids. It may serve as backbones that are essential precursors in the xanthophyll cycle. This cluster is predicted to encode a bifunctional enzyme containing domains associated with carotenoid biosynthesis. The gene with main biosynthesis functions (contig_70.g8712) encodes lycopene cyclase domains, which is a family that includes neoxanthin synthase, a crucial enzyme involved in the synthesis of neoxanthin from violaxanthin (79). It was shown in higher plants that lycopene cyclase and neoxanthin synthase exhibit extremely high similarities in protein sequences and function (80). The biosynthetic-additional gene (contig_70.g8713) was identified as phytoene desaturase, which is responsible for the conversion of phytoene into lycopene (25). Furthermore, comparative analysis revealed that other genes in this cluster may also be linked to carotenoid biosynthesis and its regulation. Contig_70.g8707 encoded a GNAT family N-acetyltransferase that is a class of enzymes commonly involved in regulatory and auxiliary functions in fungal secondary metabolism, including transcription control, antioxidant cellular protection, and stress regulation (81). Contig_70.g8715, which wasn’t present in any other cluster of other annotated *A. tubingensis* strains and wasn’t classified into any of the orthogroups, was also part of this cluster. Interestingly, it showed similarity to Celp0028 effector-like protein, which could be related to virulence and stress tolerance of the fungi (82). Such identification and lack of corresponding genes in clusters of other strains suggest that contig_70.1.g8715 may have a potential auxiliary or regulatory role associated with the activation of the carotenoid biosynthetic pathway.

Cluster contig_70 – Region 1 and its genes represent a robust genetic candidate for the biosynthesis of C40 carotenoid precursors. The encoded enzyme with main biosynthetic function, annotated as a bifunctional phytoene synthase/lycopene cyclase, is well positioned to catalyze the early committed steps of carotenoid formation, leading to the production of cyclic carotenes such as β-carotene. While this enzyme does not directly mediate epoxidation or de-epoxidation reactions of the xanthophyll cycle, its activity is essential for generating the carotenoid backbone that serves as the substrate for downstream oxygenation and modification processes. These results support a central upstream role for contig_70.g8712 in contributing upstream to the biosynthetic flux toward violaxanthin, antheraxanthin, and neoxanthin. Additionally, contig_70.g8707 and contig_70.g8715 may serve as genes with regulatory functions, activating the production of xanthophyll precursors under saline stress conditions. However, no genes directly related to the biosynthesis of identified xanthophylls have been found.

The synthesis of xanthophyll pigments in microorganisms might be influenced by abiotic stress conditions, which could trigger pigment production as a defense mechanism. Factors regulating the xanthophyll cycle, so the production of violaxanthin, antheraxanthin, and zeaxanthin, include light, temperature, oxidative and osmotic stress, as well as nutrient availability (83, 84).

In the present study, the Taguchi experimental design evaluated pH, temperature, salinity, and aeration, demonstrating that xanthophyll synthesis in *A. tubingensis* FF14 is maximized under optimal and only slightly stressful conditions. Furthermore, preliminary results suggested pigment accumulation in the absence of light; experiments were only conducted in the dark, excluding light exposure as an experimental factor. Our study indicated that temperature and salinity were the most influential factors of pigment production, together accounting for more than 60% of the observed contributions. Both factors showed the highest pigment yield in optimal or close to optimal growth conditions for strain FF14 (30°C and 5% NaCl concentration). Temperature is one of the most important environmental factors influencing both growth and biosynthetic pathways (85). Temperature can differentially influence the production of individual carotenoids depending on the strain and carotenoid tested. *Mucor circinelloides* MS12 produced the highest levels of canthaxanthin and echinenone at 20°C, while the optimal temperature for β-carotene production was 35°C (86). Additionally, in *Sporobolomyces pararoseus* CJR, various carotenoids were affected differently in response to low- and high-temperature stress. Torulene and β-carotene exhibited the highest production rates at optimal temperature conditions (25°C). In contrast, production of torularhodin significantly decreased at low temperatures and increased at high temperatures (87). On the other hand, reports on *Knufia petricola* A95 show that different temperatures, such as 25°C and 4°C, may not alter carotenoid concentrations (88). Sodium chloride was also shown to influence production of carotenoids in microorganisms. Preliminary studies have shown that salt influences the orange pigmentation of *A. tubingensis* FF14. The absence of NaCl and extremely high salt concentrations result in reduced pigment accumulation in the mycelia, while optimal to slightly elevated salt concentrations lead to the highest levels of pigment accumulation. When designing the Taguchi model, we aimed to confirm these findings. A similar correlation was noticed for *Cordyceps militaris* CM10, which showed the highest peak for carotenoid production at 2 g/L NaCl with significantly decreased production under lower and higher salt concentrations (89). For *Rhodosporidium toruloides,* a 10% increase in carotenoid production was observed under osmotic stress due to a 10% NaCl concentration (90). In the case of *S. pararoseus* NGR, a similar correlation with temperature can be observed, as varying salt concentrations influence the production of β-carotene, torulene, and torularhodin differently (91). The optimal salt concentrations for the accumulation of these compounds were 0 M, 0.75 M, and 1.0 M NaCl, respectively.

Our findings suggest that *A. tubingensis* FF14 only produces xanthophylls under conditions that favor its growth, as temperature and osmotic stress reduce its production potential. Interestingly, larger volumes of the growth media resulted in increased pigment production, indicating that reduced aeration, and potentially lower oxygen availability, may act as a mild stress factor on pigment accumulation.

The Taguchi experiment indicated that *A. tubingensis* FF14 is a promising producer of a mixture of xanthophylls, especially as a wild-type strain, compared to other sources of violaxanthin, neoxanthin, and antheraxanthin. In the optimized experiment, production of xanthophyll mixture was 39.74 ± 7.92 mg/g of wet biomass, while pigment productivity reached 2.00 ± 0.35 g/L of culture. In our study, we determined the total intracellular pigment extract content rather than individually quantified xanthophylls. This differs from most studies reporting specific compounds expressed as dry cell weight (DCW) or culture volume. As a result, direct, quantitative comparison with literature data is limited. Therefore, the obtained results should be considered an indicator of the overall pigment production potential rather than a precise measure of the biosynthetic efficiency of individual xanthophylls. An engineered strain of *Saccharomyces cerevisiae* produced violaxanthin at a yield of 7.3 mg/g DCW (92). The microalgal mutant *N. oceanica* WS-1 M1 achieved a violaxanthin yield of 5.21 ± 0.34 mg/g, with a corresponding productivity of 10.08 ± 0.66 mg/L (93). Additionally, the metabolically engineered *Escherichia coli* demonstrated a violaxanthin production of 25.28 ± 3.94 mg/g DCW (73). Different studies on engineered *E. coli* for *de novo* neoxanthin biosynthesis reported neoxanthin production of 37.3 mg/L (94). The pigment mixture extracted from *N. oceanica* NMBluh014 contained 6.6 mg of violaxanthin and 120 μg of antheraxanthin per gram of DW, along with chlorophyll a and zeaxanthin (95). To date, there is no industrial-scale production of the xanthophylls identified in this study (violaxanthin, antheraxanthin, and neoxanthin). The reported production yields of these pigments remain insufficient to support commercial feasibility (73). Moreover, no plant sources capable of accumulating violaxanthin, antheraxanthin, or neoxanthin at economically viable levels have been identified (96). Consequently, microorganisms are considered as promising alternative producers to address this limitation (93, 94).

The xanthophyll pigment mixture produced by *A. tubingensis* FF14 exhibited measurable antioxidant activity, significantly reducing DPPH and showing antioxidant capacity via FRAP assay. Although this activity was significantly lower than those of pure xanthophylls. Interpretation of oxidative stress data requires caution because this study compares a single, well-defined carotenoid (β-carotene) with a multicomponent extract. Such extracts may contain different carotenoids and other bioactive molecules, each of which may contribute to the overall antioxidant response. Therefore, direct comparisons of data between a purified standard and a complex extract should be conducted with caution. Due to the overlapping presence of several carotenoids within the extract, establishing a direct quantitative correlation between individual compounds and the measured antioxidant capacity was not feasible. Nevertheless, based on the relative abundance of the detected pigments and their known antioxidant properties reported in the literature (97), it is likely that all three carotenoids contribute to the overall activity.

The activity to scavenge 50% of DPPH radicals for purified violaxanthin extracted from the microalga *Eustigmatos cf. polyphem* was 41.42 μg/mL (64). In contrast, the pigment extract from *Flavobacterium* sp. JSWR-1, which primarily contained zeaxanthin, exhibited IC_50_ values of 0.933 and 1.75 mg/mL, indicating a lower antioxidant potential compared to the pure zeaxanthin standard (IC_50_ = 82.74 µg/mL) (98). The xanthophyll pigment from *Erythrobacter* sp. SDW2, identified as a zeaxanthin derivative, showed an IC_50_ value of 13.2 mg/L for DPPH (36), nearly four times lower than the DPPH activity observed in this study.

Results were compared to the scavenging activity of the β-carotene standard, indicating that while the xanthophyll pigment mixture showed lower values, it still exhibited high and significant antioxidant activity. Although the pigment’s scavenging ability was lower than that of the positive control, it consistently exhibited significant potential for scavenging DPPH radicals. These results suggest that the pigment obtained from *A. tubingensis* FF14 shows notable antioxidant activity, although lower than the β-carotene standard.

Although zeaxanthin was not produced in this study, the metabolic pathways and presence of its precursors suggest that the production of this compound is possible. Zeaxanthin is a natural antioxidant and colorant with significant applications in the food, nutraceutical, and pharmaceutical industries, particularly in the prevention of age-related eye diseases (99–101). Additionally, it has potential as a food additive due to its antioxidant properties and as a dye in the textile industry (102, 103). Future studies will focus on modulating production conditions to obtain this compound.

### Limitations of the study

This study was unable to unambiguously identify the specific genes directly responsible for xanthophyll biosynthesis in *Aspergillus tubingensis* FF14. Despite conducting comparative analyses with other *A. tubingensis* genomes, including orthology-based comparison of genes and biosynthetic gene cluster similarity analysis, no unique genes or BGCs linked to xanthophyll production were identified. However, preliminary analyses suggest that carotenoid and xanthophyll biosynthesis may be related to differential regulation of conserved gene clusters or to sequence variation within genes already present in *A. tubingensis*. Determining the precise genetic mechanisms underlying xanthophyll production will require transcriptomic analyses under pigment-inducing conditions.

### Conclusions

This study presents the first report of *Aspergillus tubingensis* producing xanthophylls and expands the metabolic potential of widely distributed *Aspergillus* species. Moreover, there are no previous reports of environmental isolates of *Aspergillus* from extremophilic habitats producing pigments from the carotenoid group. An insight is given into pigment production by fungi, which is promising from a biotechnological perspective, given the need for sustainable and biological sources of carotenoids. The obtained pigments were identified as a mixture of three xanthophylls (violaxanthin, antheraxanthin, and neoxanthin), and their antioxidant potential and improved production conditions were here defined. Based on the identification and the identified properties, the obtained pigment mixture could be used in the food industry as colorants, in healthcare as nutraceuticals, or in cosmetics as UV-protective agents (65, 104, 105).

Genomic analyses provided further insight into the biosynthetic gene cluster that is potentially linked to carotenoid biosynthesis. However, the specific mechanisms responsible for xanthophyll biosynthesis in *A. tubingensis* FF14 remain to be described. The genome annotation that has been achieved so far has not made it possible to reconstruct the mechanism and trace the pathway by which the fungus produces these pigments. The annotation of fungal genes, in general, certainly has gaps, especially compared to that of more studied model organisms. These gaps can be filled through an analysis dedicated to knowing the genes transcribed under certain conditions, including those that promote the production of these interesting pigments.

## MATERIALS AND METHODS

### Fungal strain

The studied fungal strain FF14 is a halophilic strain isolated from a site in the Burano area of Grosseto, Italy (42°23′43.7″N, 11°24′40.0’″E) in October 2019. The fungus was isolated from coastal soils that are periodically penetrated by seawater, leading to natural salinization. The strain was initially identified as *Aspergillus neoniger* based on ITS and TEF1-α nucleotide sequences and deposited in the NCBI database with accession numbers PQ596437 and PQ644624, respectively. The strain was inoculated on MEA plates and incubated for 7 days at 30°C, then stored at 4°C for further use.

### Chemicals and standards

The β-carotene pigment standard was purchased from Angene Chemical. Analytical solvents for extraction and UHPLC analysis, including HPLC-grade solvents such as methanol, acetonitrile, acetone, and ethanol, were sourced from Chempur and J.T. Baker.

The chemicals used for assessing antioxidant activity were 2,2-diphenyl-2-picrylhydrazyl hydrate (DPPH), 2,4,6-tris(2-pyridyl)-s-triazine (TPTZ), ferric chloride, ferrous sulfate heptahydrate, and ascorbic acid, which were acquired from Angene and Merck.

### Culture conditions for pigment production

*A. tubingensis* FF14 was cultured on a ME medium (malt extract, 30 g/L; peptone, 5 g/L; pH 5.4) supplemented with 5% (wt/vol) NaCl for 10 days at 30°C, 120 rpm. The microorganism cultivation was carried out in 250 mL Erlenmeyer flasks containing 50 mL sterilized broth. After the incubation period, fungal biomass was collected by filtering the culture broth through a Büchner funnel. The fungal biomass was washed and separated to determine wet weight and then frozen at −20°C until use.

### Pigments extraction

Frozen wet biomass was weighed, and 10 times the volume of acetone/methanol (7:3, vol/vol) was added (10 mL per each 1 g of biomass). To extract intracellular pigments, biomass was ground in an organic solvent solution in a mortar for 5 min. In the next step, the obtained biomass extract was transferred into a screwed tube, and extraction was carried out by shaking for 2 h at 60°C at 200 rpm (106), in the dark. The residues of biomass were removed by centrifugation at 10,000 rpm for 15 min at 4°C. The obtained supernatant was stored at −20°C until analysis of pigments.

### Pigment identification and analysis

The extracted pigments were analyzed to determine their chemical nature, concentration, and free radical scavenging activity. This was achieved by ultraviolet-visible spectrophotometry (UV-Vis), UHPLC-DAD-ESI-MS/MS, and DPPH and FRAP antioxidant assays.

### Ultraviolet-visible spectroscopy

UV-Vis spectra were recorded using a MultiScanGo plate reader (Thermo Fisher Scientific, Waltham, MA, USA) in the range of 200 nm–700 nm. β-Carotene was tested as a standard. The organic solvents used for extraction were then used as blanks.

### UHPLC-DAD-ESI-MS/MS analysis

UHPLC-DAD-ESI-MS analysis was performed to determine the carotenoid profile of the obtained pigment extracts. The samples were dried under a gentle stream of nitrogen and re-dissolved in methanol. UHPLC-DAD analysis was performed using a Dionex UltiMate 3000 high-resolution UHPLC liquid chromatograph (Thermo Fisher Scientific Inc., Waltham, MA, USA), equipped with a diode array detector (DAD) (Thermo Fisher Scientific Inc., Waltham, MA, USA). UHPLC-ESI-MS/MS analysis was performed using a Q Exactive Hybrid Quadrupole-Orbitrap mass spectrometer (Thermo Fisher Scientific Inc., Waltham, MA, USA), a high-resolution mass spectrometry system coupled with a Transcend TLX-1 high-resolution UHPLC liquid chromatograph (Thermo Fisher Scientific Inc., Waltham, MA, USA). The Accucore C30 column (2.6 × 100 mm, 3 µm) was used for separation. The following separation parameters were used: column temperature: 40°C; mobile phase flow: 0.3 mL/min; isocratic elution; mobile phase A: methanol; sample injection volume: 10 µL. Spectrophotometric detection was performed at different wavelengths: 220, 280, 320, and 450 nm. Chromeleon 6.8.1 Chromatography Data System software (Thermo Fisher Scientific Inc., Waltham, MA, USA) was used to control, record, and analyze the obtained results. ESI‑MS/MS analysis was performed under the following conditions: capillary voltage of 5,000 V in positive ion mode; capillary temperature of 280°C; drying gas temperature of 350°C. Nitrogen was used as the shielding and auxiliary gas at flow rates of 50 and 14 (arbitrary units), respectively. Data were acquired in parallel reaction monitoring (PRM) operating mode with a collision energy of 15 eV and a mass scan range of 100–1,500 m/z. MS/MS fragmentation spectra were obtained in collision-induced dissociation mode using nitrogen as the collision gas. Q Exactive Tune 2.1, Aria 1.3.6, and Thermo Xcalibur software were used to control, record, and analyze the obtained results.

### Genome sequencing, assembly, and annotation

Biomass was cultured under the same conditions as described in the “Culture conditions for pigment production” section. Following cultivation, the biomass was harvested by vacuum filtration using a Büchner funnel. The collected biomass was then transferred to a pre-cooled mortar. Liquid nitrogen was poured over the biomass to rapidly freeze the sample, ensuring preservation of cellular integrity and facilitating subsequent homogenization. Once fully frozen, the biomass was ground into a fine powder using a pestle under continuous cooling with liquid nitrogen to prevent thawing during the homogenization process.

Genomic DNA was extracted using standard phenol-chloroform and cetyltrimethylammonium bromide methods (107). DNA was quantified with Qubit DNA Broad Range assay (Life Technologies, USA) and NanoDrop (Thermo Fisher Scientific, Waltham, MA, USA), while DNA quality was checked by agarose gel electrophoresis. The sequencing library was constructed with VAHTS Universal Plus DNA Library Prep Kit for Illumina V2 (Vazyme, China). Briefly, 500 ng of quality-controlled genomic DNA (A260/280 = 1.8–2.0) was sheared to 250 bp–350 bp fragments using a Covaris S220 focused ultrasonicator (PerkinElmer, USA) and processed with the kit. After end repair and adapter ligation, libraries were amplified with eight cycles of PCR and analyzed with LabChip GX Touch (Revvity, USA) to ensure the quality of the constructed library. Then, the QC-treated libraries were sequenced on a Salus Pro platform (Salus Bio, China) (150 bp paired-end) according to manufacturer’s instructions.

Reads were quality trimmed of adapters with Trimmomatic (v.0.39) (108). The quality of the raw reads was verified by FastQC (v.0.12.1) (109). The short reads from the two libraries were assembled by MEGAHIT (v.1.2.9) (110). Genome assembly was also carried out by SPAdes (v.4.1.0) (111) but based on the quality of the initial assembly obtained, the MEGAHIT assembler was then chosen for further analysis. The contigs were polished using RagTag (v.2.1.0) (112) and Pilon (v.1.20) (113). The quality of the obtained assembly was evaluated using QUAST (v.5.3.0) (114), while completeness was assessed with BUSCO (v.5.8.0) (115) based on the eurotiomycetes_odb10 database.

A phylogenetic tree of *Aspergillus* species was constructed using assembled whole-genome sequences. The tree was generated with REALPHY (v1.13) (116) based on reference-guided alignment and default settings. Visualization was performed using iTOL (v6) (117). Average nucleotide identity (ANI) alignment of the whole genome was performed with FastANI (v.1.3) (118), and genomic distance was calculated by RefSeq Masher (v.0.1.2) (119).

Gene prediction was performed using AUGUSTUS software (v.3.5.0) (120) with *Aspergillus oryzae* as a reference. Then, functional annotations were performed using InterProScan (v.5.59-91.0) (121), UniProt (122), eggNOG (v.2.1.13) (123), BlastKOALA (v.3.1) (124), and PANNZER2 (125). Carbohydrate-active enzyme annotation was performed using dbCAN3 server (v.13) (126). The identification of the secondary metabolite biosynthetic gene cluster was performed with antiSMASH (v.8.0.4) (127).

### Comparative genomic analysis of biosynthetic gene clusters and orthologous genes

A total of 22 *Aspergillus tubingensis* strains with whole-genome sequence were obtained from NCBI database. Protein sequences for each tested strain were generated using AUGUSTUS (v.3.5.0), with *Aspergillus oryzae* as a reference. Furthermore, biosynthetic gene clusters were identified using the web version of antiSMASH (v.8.0.4) with relaxed settings and all extra features on.

Comparative analysis of BGCs was performed using BiG-SCAPE (v.2.0.3) (128). Clustering was conducted in global (“mix”) mode with singleton inclusion to group BGCs into gene cluster families based on domain composition and sequence similarity. The comparative gene cluster analysis was conducted with Clinker using CAGECAT (v.1.0) (129, 130).

Orthologous relationships between genes were identified using OrthoFinder (v.2.5.5) (131). Unified protein sequences generated from AUGUSTUS were analyzed using default settings. The resulting orthogroups were used to assess conserved and strain-specific gene content.

### Xanthophylls production optimization

The Taguchi method was used to improve the process of pigment production by *A. tubingensis* FF14 strain. The experiment was designed to determine the conditions under which the mentioned strain produced the highest amount of identified pigments. Production of pigments was obtained based on pigment units per gram of biomass and amount of fungal biomass per milliliter of culture. The response of the system was defined as g of the mixture of xanthophylls per 1 L of culture. Design of experiments included 16 experimental setups, testing the effect of four factors on four different levels (Tables S5 and S6). The factors considered were pH (factor A), temperature (factor B), concentration of NaCl (factor C), and medium volume (factor D). The levels for pH were 3, 5, 7, and 9; for temperature, 20°C, 25°C, 30°C, and 35°C; for NaCl concentration, 0%, 5%, 10%, and 15%; and for medium volume, 15%, 20%, 30%, and 40%. Each experimental setup was performed in triplicate. All variants were grown on ME medium at 120 rpm without light access.

The R program (version 4.4.2) was used for analyzing data obtained from the Taguchi optimization experiment. Parameters were designed to calculate S/N ratios for larger-is-better desired values and generate main effects plots. ANOVA was carried out to determine the significance of the obtained results and analyze the contribution of factors.

### Antioxidant activity

The antioxidant properties of pigment derived from *A. tubingensis* FF14 were evaluated using DPPH and FRAP radical scavenging assays, with β-carotene serving as a positive control. Both pigments were initially dissolved in ethanol to create a stock solution of 10 mg/mL, followed by a twofold serial dilution series (from 5.00 to 0.078125 mg/mL) for the antioxidant tests.

For the DPPH radical scavenging assay (64), 200 µL of a 0.1 mM DPPH ethanol solution was mixed with 100 µL of the pigment solution, and the absorbance was measured at 517 nm after a 30 min incubation in the dark. Additionally, the pigment solutions were measured at 517 nm to account for any interference that might occur prior to adding the testing solution (132), with ethanol used as the blank control. The scavenging activity was calculated using the formula: DPPH inhibition [%] = [(A_blank_ – A_sample_) / A_blank_] × 100, where A_blank_ is the absorbance of the blank control, and A_sample_ is the absorbance of the test reaction adjusted for pigment absorbance.

For the FRAP assay (133), 180 µL of FRAP working solution was combined with 10 µL of the sample, followed by incubation at 37°C for 15 min in the dark, and measurement at 593 nm. The FRAP working solution was freshly prepared before the experiment by mixing acetic acid buffer (0.3 M, pH 3.6), TPTZ solution (10 mM), and FeCl_3_ solution (20 mM) in a 10:1:1 ratio. Samples were diluted with ethanol, with ethanol also serving as the blank control. Similar to the DPPH method, samples diluted with water instead of the working solution were measured at 593 nm to avoid interference with color absorbance. A solution of FeSO_4_·7 H_2_O served as the standard to obtain a calibration curve, based on which the FRAP value (μmol Fe²^+^/mL) was calculated.

## Data Availability

This Whole Genome Shotgun project has been deposited at DDBJ/ENA/GenBank under the accession JBNSKI000000000. The version described in this paper is version JBNSKI010000000.

## SUPPLEMENTAL MATERIAL

Supplemental figures and tables spectrum.01179-26-s0001.docx Figures S1 to S7 and Tables S1 to S8.

ASM does not own the copyrights to Supplemental Material that may be linked to, or accessed through, an article. The authors have granted ASM a non-exclusive, world-wide license to publish the Supplemental Material files. Please contact the corresponding author directly for reuse.
