# Supplementary material for: Genomic insights and biotechnological potential of a saline soil fungus Aspergillus tubingensis FF14 for xanthophyll biosynthesis
Source: Microbiol Spectr. 2026 May 18;14(7):e01179-26. doi: 10.1128/spectrum.01179-26 (PMC13340296; doi:10.1128/spectrum.01179-26)
Supplement: Supplemental figures and tables — Figures S1 to S7 and Tables S1 to S8. [file spectrum.01179-26-s0001.docx]

Genomic insights and biotechnological potential of a saline soil fungus *Aspergillus tubingensis* FF14 for xanthophyll biosynthesis

Weronika Śliżewska^1,*^, Katarzyna Struszczyk-Świta^1^, Joanna Oracz^2^, Flavia Pinzari^3,4^, Maurycy Daroch^5^, Agata Czyżowska^6^ and Olga Marchut-Mikołajczyk^1,*^

1 Institute of Molecular and Industrial Biotechnology, Faculty of Biotechnology and Food Sciences, Lodz University of Technology, Lodz, Poland

2 Institute of Food Technology and Analysis, Faculty of Biotechnology and Food Sciences, Lodz University of Technology, Lodz, Poland

3 National Research Council of Italy (CNR), Institute for Biological Systems, Rome, Italy

4 Natural History Museum, London, UK

5 School of Environment and Energy, Peking University Shenzhen Graduate School, Shenzhen, China

6 Institute of Fermentation Technology and Microbiology, Faculty of Biotechnology and Food Sciences, Lodz University of Technology, Lodz, Poland

* Correspondence:

weronika.slizewska@dokt.p.lodz.pl (WS); olga.marchut-mikolajczyk@p.lodz.pl (OMM)

Keywords: halophilic fungi; *Aspergillus tubingensis*; secondary metabolites; xanthophylls; genomics

**Table S1** The identity comparison of strain FF14 with reference *Aspergillus* species based on Average Nucleotide Identity (ANI) percentage generated by FastANI and identity values generated by RefSeq Masher.

| Reference strain | Accession Number | ANI*  (%) | RefSeq Masher | |
| --- | --- | --- | --- | --- |
|  |  |  | Identity  (%) | Shared hashes* |
| *Aspergillus* *tubingensis* WU-2223L | GCA_013340325.1 | 97.95 | 100 | 400/400 |
| *Aspergillus* *costaricaensis* CBS 115574 | GCA_003184835.1 | 96.05 | 100 | 400/400 |
| *Aspergillus* *neoniger* CBS 115656 | GCA_003184625.1 | 95.81 | - | - |
| *Aspergillus* *piperis* CBS 112811 | GCA_003184755.1 | 94.59 | 99.87 | 392/400 |
| *Aspergillus* *niger* CBS 513.88 | GCA_000002855.2 | 89.17 | 99.40 | 363/400 |
| *Aspergillus* *awamori* IFM 58123 | GCA_003850985.1 | 89.10 | 99.45 | 366/400 |
| *Aspergillus* *brasiliensis* CBS 101740 | GCA_001889945.1 | 87.47 | 98.65 | 322/400 |

*ANI is defined as mean nucleotide identity of orthologous gene pairs shared between two microbial genomes (1); hashes refer to compressed representations of k-mers derived from genomic sequences using the MinHash algorithm, enabling efficient estimation of sequence similarity by quantifying the number of shared hashes between query and reference datasets (2).


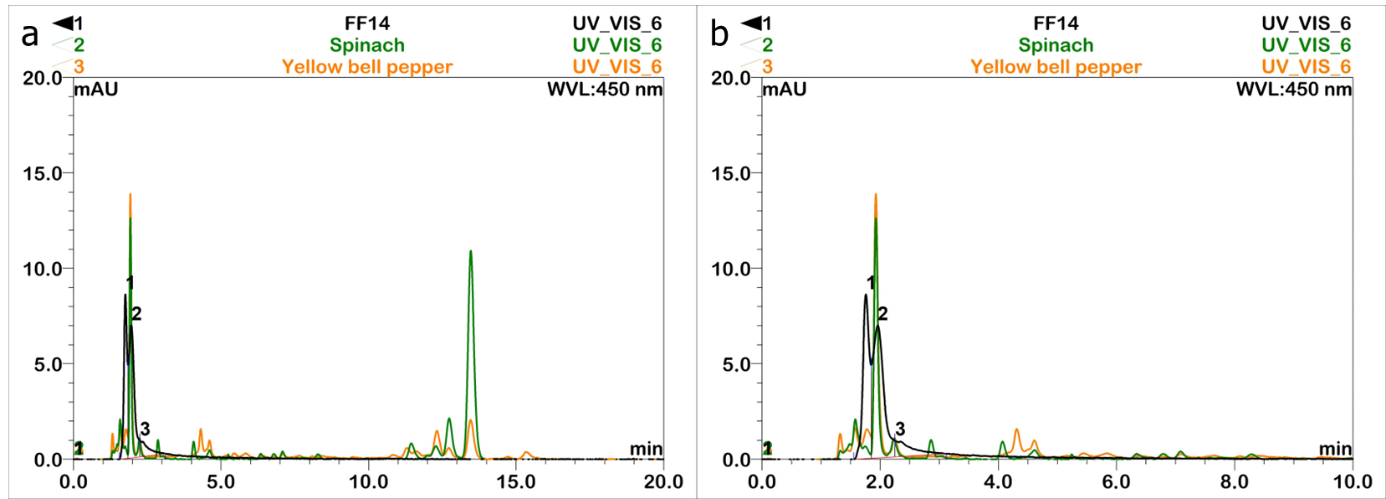


**Figure S1** UHPLC-DAD spectra of pigment of *A. tubingensis* FF14 in comparison with extracts of yellow bell pepper and spinach. a) Full HPLC profile (25 minutes) for 450 nm. b) Partial HPLC spectrum for 10 minutes to better show early peaks for 450 nm.


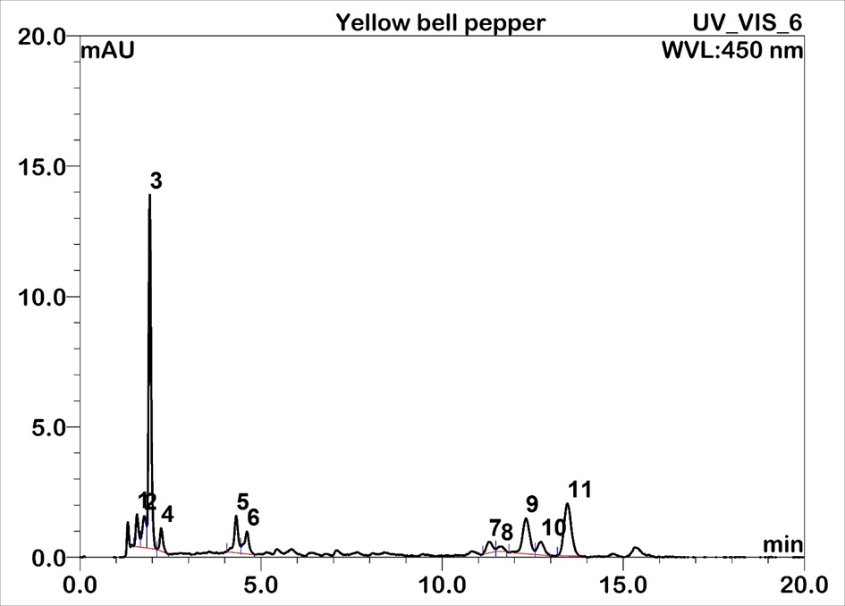


**Figure S2** UHPLC-DAD chromatogram of carotenoids in yellow bell peppers. Peak identification: 1 - (E)-neoxanthin, 2 - (Z)-neoxanthin, 3 - (E)-violaxanthin, 4 - (Z)-anteraxanthin, 5 - (E)-lutein, 6 - (Z)-lutein, 7- α-cryptoxanthin, 8 - β-cryptoxanthin, 9 - ζ-carotene, 10 -α-carotene, 11 - β-carotene.


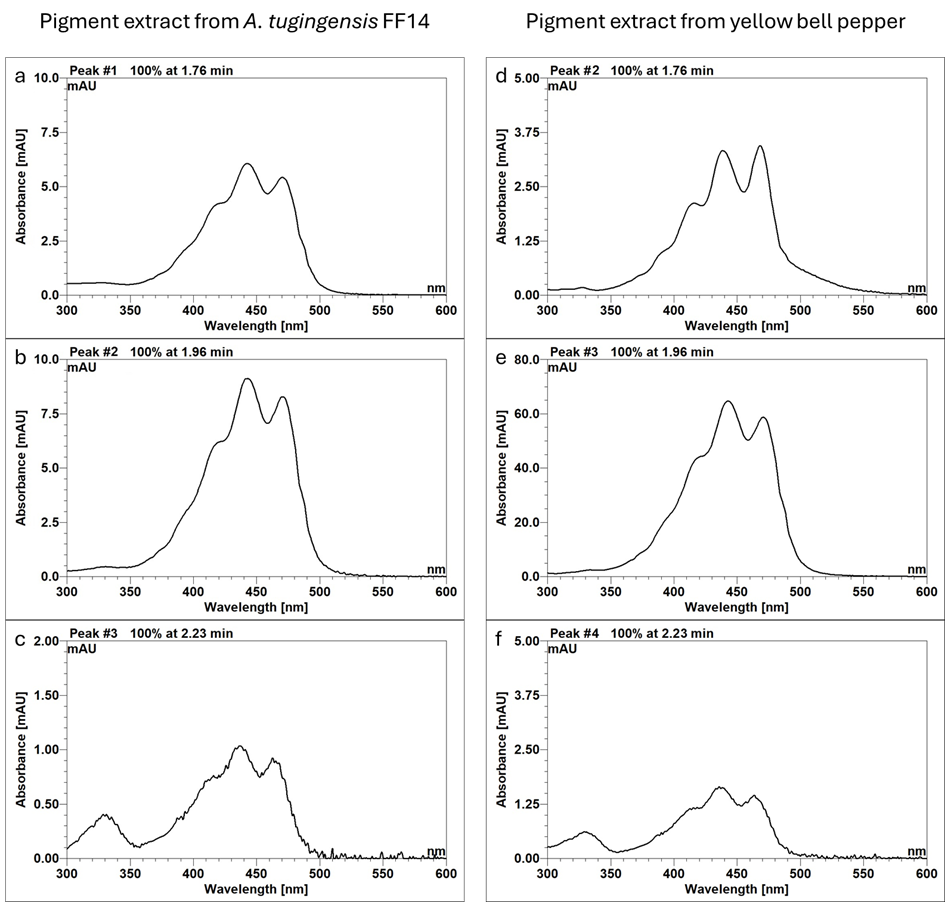


**Figure S3** Comparison of UV-vis spectra obtained from a,b,c) FF14 pigment extract and d,e,f) yellow bell pepper extract, for neoxanthin (a,d), violaxanthin (b,e), and antheraxanthin (c,f).


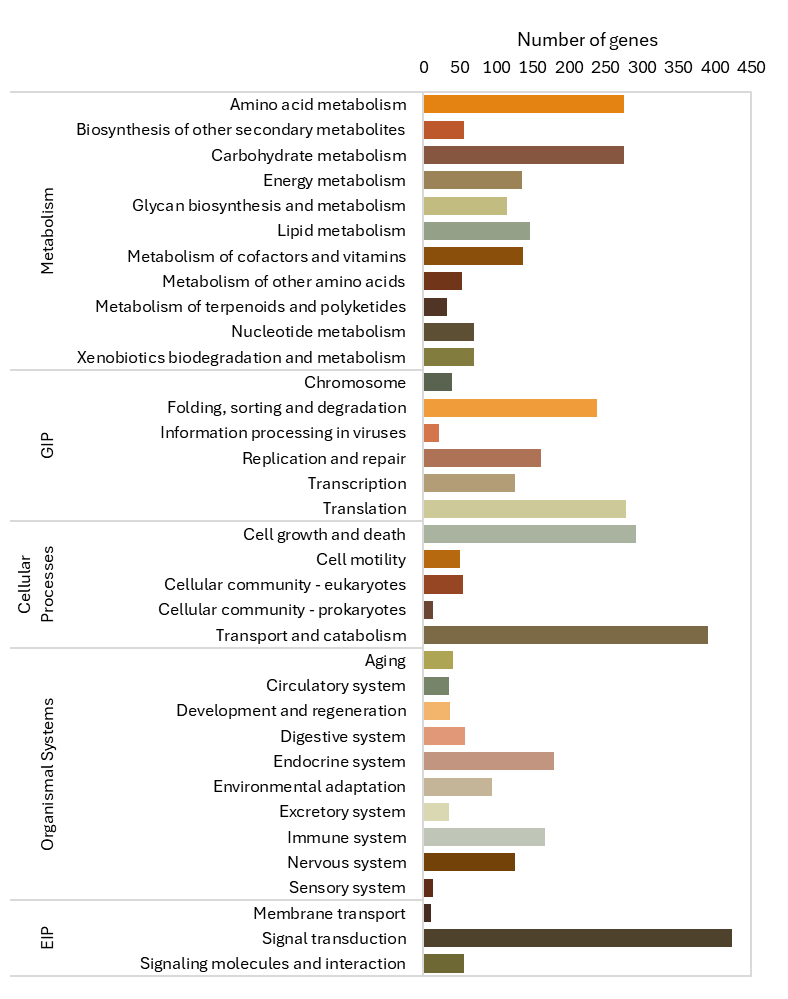


**Figure S4** Results of KEEG pathway analysis for *A.tubingensis* FF14. GIP: Genetic Information Processing; EIP: Environmental Information Processing.


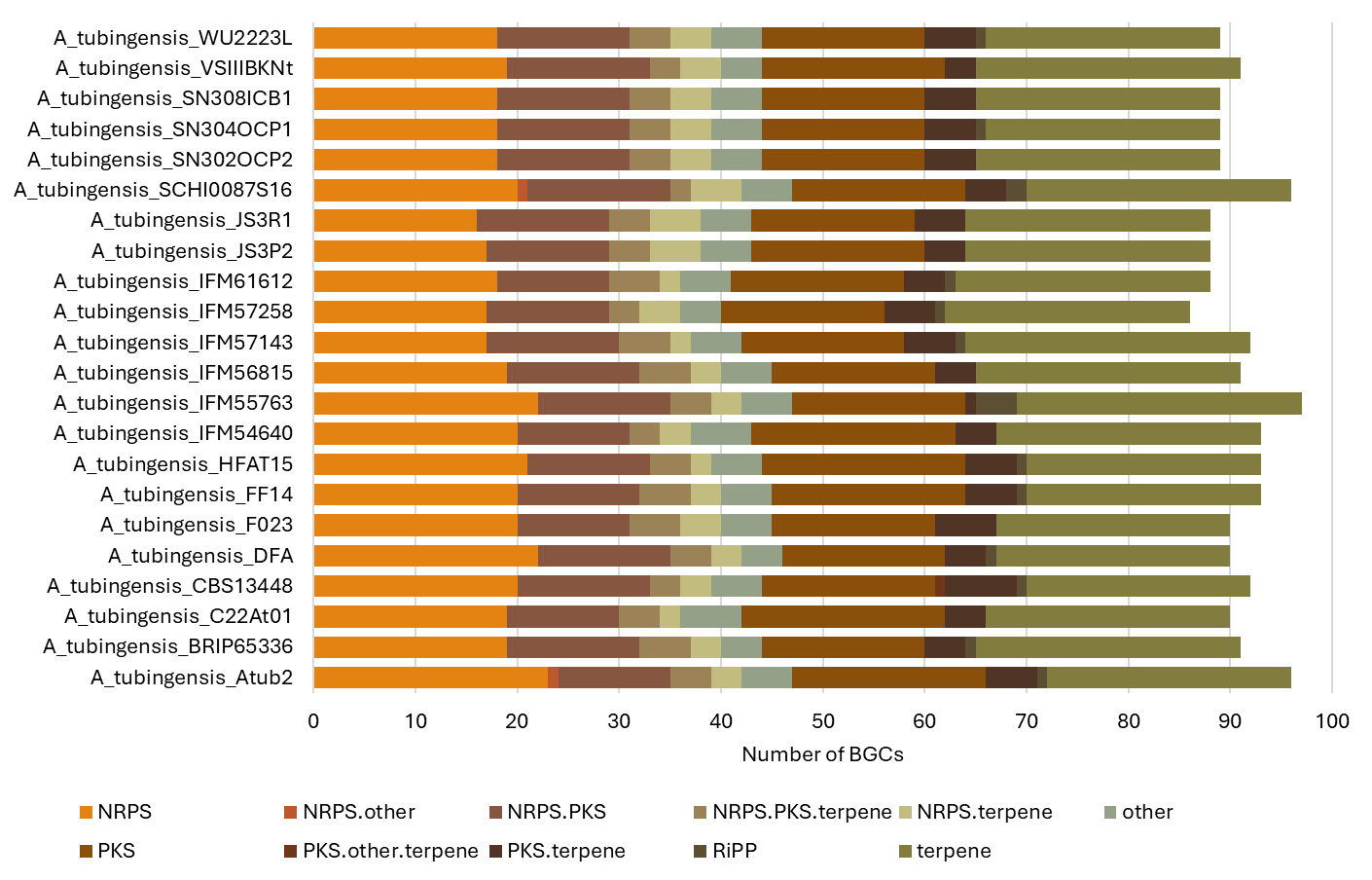


**Figure S5** The number and types of secondary metabolite BGCs in *A. tubingensis* FF14 and other closely related *A. tubingensis* strains deposited in antiSMASH database.

**Table S2** BGCs prediction by fungiSMASH8 on the genome of *A. tubingensis* FF14

| **Contig** | **Cluster** | **Type** | **Length** | **Similarity Confidence** | **Most similar known cluster** |
| --- | --- | --- | --- | --- | --- |
| 4 | 1 | NRPS-like, T1PKS | 100,055 | High | choline |
| 5 | 3 | T1PKS | 67,408 | Low | sordarial |
| 8 | 1 | terpene | 32,317 | High | clavaric acid |
|  | 3 | T1PKS | 66,668 | High | YWA1 |
|  | 5 | NRPS-like | 63,095 | Medium | kojic acid |
| 10 | 1 | NRPS | 65,971 | Low | notoamide F/(+)-semivioxanthin/waikikiamide B/waikikiamide C/notoamide A/waikikiamide A |
| 11 | 3 | terpene, NRPS, T1PKS | 186,061 | Low | azanigerone A/azanigerone B/azanigerone C/azanigerone D/azanigerone E/azanigerone F |
| 12 | 1 | NRPS-like, T1PKS | 76,783 | Low | verrucosidin |
| 14 | 3 | T1PKS, NRPS-like | 117,657 | Low | HEx-pks23 polyketide |
| 17 | 3 | T1PKS, terpene | 99,357 | Low | ankaflavin/monascin/rubropunctatine/monascorubrin |
| 21 | 1 | T1PKS | 114,594 | High | TAN-1612/1-(2,3,5,10-tetrahydroxy-7-methoxy-4-oxo-1,2,3,4-tetrahydroanthracen-2-yl)pentane-2,4-dione/desmethyl TAN-1612 |
| 22  27 | 2 | terpene, NRPS, T1PKS | 101,128 | Medium | pyranoviolin A |
|  | 3 | NRPS, T1PKS | 68,211 | High | AbT1 |
| 32 | 2 | T1PKS, terpene | 90,304 | Medium | epipyrone A |
| 33 | 2 | terpene | 32,491 | High | sesterfisherol/sesterfisheric acid/compound 6/compound 7a/compound 5/compound 8 |
| 49 | 2 | terpene-precursor, NRPS, T1PKS, terpene | 93,254 | Low | 14-(N,N-dimethylleucyloxy)paspalinine/14-(leucyloxy)paspalinine/14-hydroxypaspalinine |
| 52 | 1 | NRPS-like, T1PKS | 43,371 | High | pyrophen/campyrone B |
| 63 | 1 | T1PKS | 80,928 | Low | 4-epi-15-epi-brefeldin A |
| 67 | 2 | terpene | 23,318 | Low | 14-(N,N-dimethylleucyloxy)paspalinine/14-(leucyloxy)paspalinine/14-hydroxypaspalinine |
| 102 | 1 | NRPS | 51,867 | Low | nidulanin A |


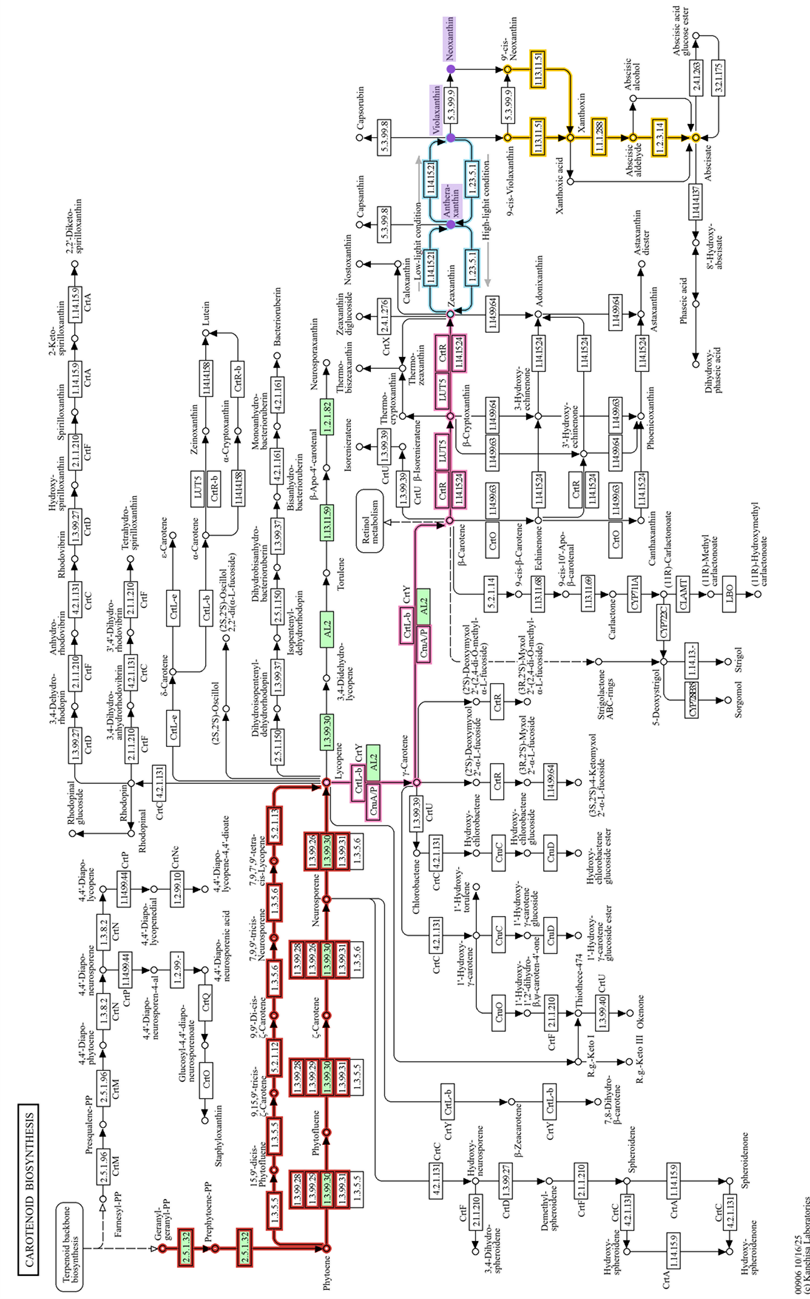


**Figure S6** Genes encoding carotenoids metabolism identified in *A. tubingensis* FF14. Biosynthesis pathways were marked: lycopene biosynthesis (in red), zeaxanthin biosynthesis (in pink), xanthophyll cycle (in blue), and abscisic acid biosynthesis, plants (in yellow). In green genes found in tested strain using KEGG. In purple pigments produced by tested strain, identified in this study were marked.

**Table S3** Genomes of *A. tubingensis* strains used in comparative analysis

| **Organism** | **Strain code** | **GenBank assembly accession number** | **Country of origin** | **Isolation source** |
| --- | --- | --- | --- | --- |
| *Aspergillus tubingensis* | Atub2 | GCA_900163765.1 | France | Vineyard |
|  | BRIP 65336 a | GCA_044592785.1 | Australia | Unknown |
|  | C2-2 At_01 | GCA_010614855.1 | South Korea | Leaven |
|  | CBS 134.48 | GCA_001890745.1 | Unknown | Unknown |
|  | DFA | GCA_049803905.1 | USA | Moldy date palm fruit |
|  | F023 | GCA_040333235.1 | China | *Acanthus ilicifolius* |
|  | **FF14** | **GCA_050924195.1** | **Italy** | **Saline soil** |
|  | HFAT15 | GCA_030762845.1 | China | Feces |
|  | IFM 54640 | GCA_027923605.1 | Japan | Human, sputum |
|  | IFM 55763 | GCA_027923625.1 | Japan | Vineyard |
|  | IFM 56815 | GCA_027923885.1 | Japan | Human ear |
|  | IFM 57143 | GCA_027923665.1 | Japan | Human |
|  | IFM 57258 | GCA_027923685.1 | Japan | Soil |
|  | IFM 61612 | GCA_027923925.1 | Japan | Human, bronchoalveolar lavage fluid |
|  | JS3-P2 | GCA_019827565.1 | USA | HEPA air filter |
|  | JS3-R1 | GCA_019827425.1 | USA | HEPA air filter |
|  | SCHI0087.S.16 | GCA_044589405.1 | Australia | Human, sputum |
|  | S/N-302-OC-P2 | GCA_019828785.1 | USA | Spx12/RR9 Outside cage |
|  | S/N-304-OC-P1 | GCA_019827465.1 | USA | Spx11/RR5 Outisde cage |
|  | S/N-308-IC-B1 | GCA_019827445.1 | USA | Spx12/RR9 Inside cage |
|  | VS III B KN t | GCA_019805365.1 | USA | Vacuum filter from cleanroom |
|  | WU-2223L | GCA_013340325.1 | Japan | Soil |


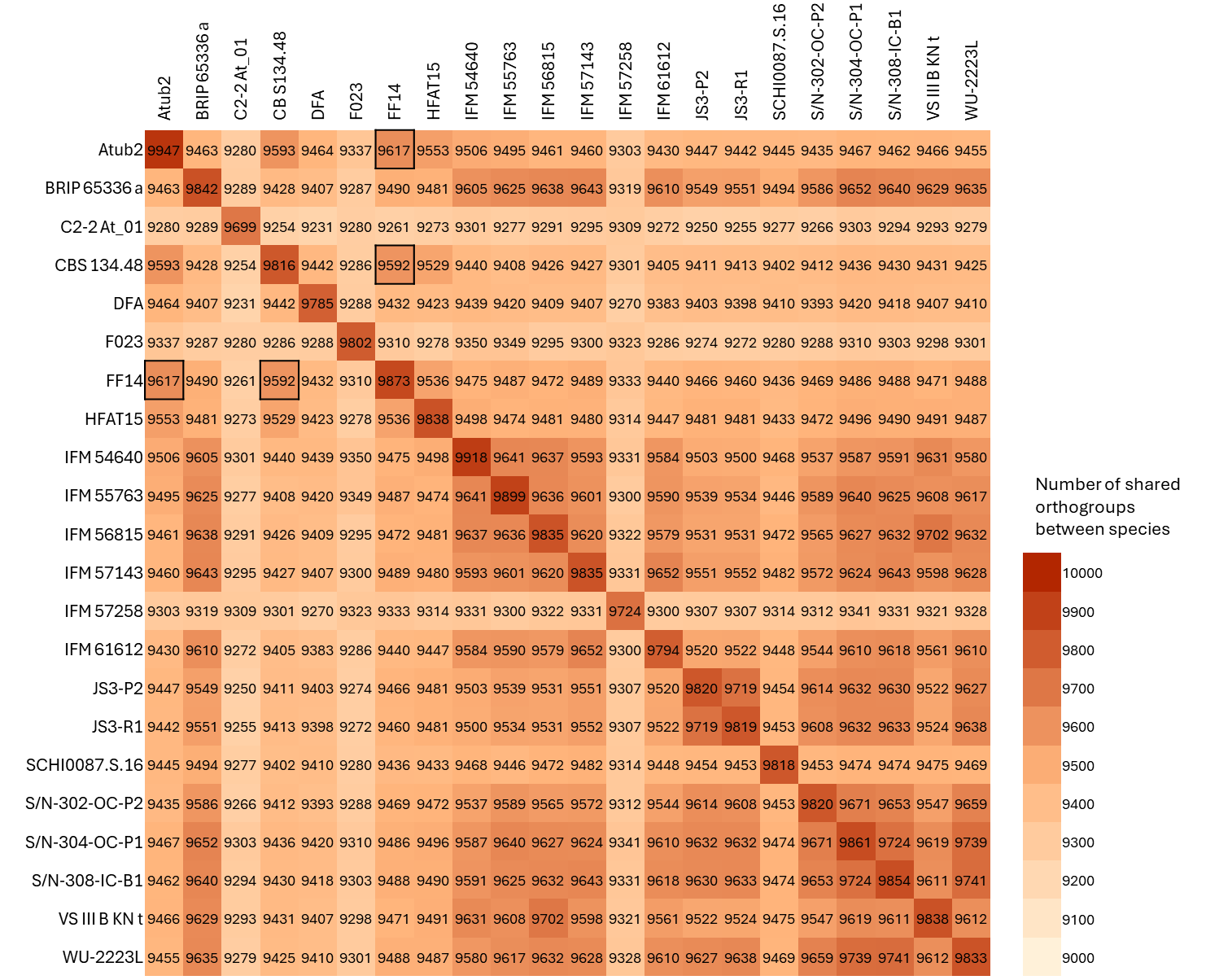


**Figure S7** Heatmap of pairwise orthogroup overlapping among *A. tubingensis* species based on OrthoFinder results. Values represent the number of shared orthogroups.

**Table S4** Identification of genes of *A.tubingensis* FF14 that were not assigned to any orthogroup during comparative analysis via Orthofinder.

| **Contig** | **Gene** | **Length** | **UniProt identification** | | | |
| --- | --- | --- | --- | --- | --- | --- |
|  |  | **AA** | **Protein name** | **Closest match organism** | **Identity [%]** | **E-value** |
| 3 | g902 | 192 | Uncharacterized protein | *Aspergillus tubingensis* (strain CBS 134.48) | 81.2 | 7.70E-93 |
| 4 | g1005 | 188 | Cyanovirin-N domain-containing protein | *Aspergillus novoparasiticus* | 72.5 | 3.30E-101 |
| 6 | g1650 | 214 | Uncharacterized protein | *Aspergillus awamori* (Black koji mold) | 55.2 | 5.00E-11 |
| 9 | g2167 | 241 | Cytochrome b561 domain-containing protein | *Aspergillus tubingensis* (strain CBS 134.48) | 99.2 | 2.20E-166 |
| 11 | g2549 | 748 | Uncharacterized protein | *Aspergillus aculeatus* (strain ATCC 16872 / CBS 172.66 / WB 5094) | 82.8 | 0 |
|  |  |  | Ankyrin repeat protein | *Coccidioides posadasii* (strain RMSCC 757 / Silveira) (Valley fever fungus) | 65.1 | 0 |
|  | g2754 | 131 | Reverse transcriptase domain-containing protein | *Aspergillus tanneri* | 67.2 | 1.80E-57 |
|  | g2755 | 260 | CCHC-type domain-containing protein | *Rasamsonia emersonii* (strain ATCC 16479 / CBS 393.64 / IMI 116815) | 34.7 | 1.60E-45 |
| 13 | g2956 | 147 | Velvet domain-containing protein | *Aspergillus welwitschiae* | 81.8 | 3.70E-73 |
| 15 | g3383 | 237 | Uncharacterized protein | *Aspergillus eucalypticola* (strain CBS 122712 / IBT 29274) | 94.1 | 5.70E-164 |
| 17 | g3816 | 188 | GMC oxidoreductase | *Canariomyces notabilis* | 47 | 1.00E-17 |
| 22 | g4514 | 169 | Uncharacterized protein | *Aspergillus sclerotialis* | 80 | 2.30E-16 |
| 30 | g5605 | 567 | GATA-type domain-containing protein | *Aspergillus tubingensis* (strain CBS 134.48) | 100 | 0 |
| 34 | g6088 | 261 | NADPH-dependent FMN reductase Lot6 | *Aspergillus neoniger* (strain CBS 115656) | 96.6 | 0 |
|  |  |  | Amine oxidase domain-containing protein | *Aspergillus kawachii* (White koji mold) (*Aspergillus awamori* var. *kawachi*) | 93.9 | 3.20E-180 |
| 64 | g8436 | 279 | C2H2-type domain-containing protein | *Aspergillus tubingensis* (strain CBS 134.48) | 99.6 | 0 |
|  |  |  | Transcriptional regulator RPN4 | *Aspergillus awamori* (Black koji mold) | 97.4 | 0 |
| 70 | g8715 | 251 | Uncharacterized protein | *Aspergillus costaricaensis* CBS 115574 | 98 | 1.50E-176 |
|  |  |  | Celp0028 effector like protein | *Aspergillus vadensis* (strain CBS 113365 / IMI 142717 / IBT 24658) | 96 | 2.80E-174 |
|  | g8753 | 871 | Carrier domain-containing protein | *Aspergillus tubingensis* (strain CBS 134.48) | 100 | 0 |
|  |  |  | Nonribosomal peptide synthase | *Aspergillus costaricaensis* CBS 115574 | 93.1 | 0 |
| 120 | g9987 | 967 | AMP-activated protein kinase glycogen-binding domain-containing protein | *Aspergillus tubingensis* (strain CBS 134.48) | 98 | 0 |
|  |  |  | PT repeat family protein | *Aspergillus vadensis* (strain CBS 113365 / IMI 142717 / IBT 24658) | 93.7 | 0 |
| 121 | g9994 | 330 | Uncharacterized protein | *Aspergillus tubingensis* (strain CBS 134.48) | 100 | 0 |
|  |  |  | calcium/calmodulin-dependent protein kinase, EC:2.7.11.17 | *Aspergillus awamori* | 71.5 | 2.80E-156 |

**Table S5** Design of experiment for Taguchi method: factors and their levels

| **Symbol** | **Factor** | **Level** | | | |
| --- | --- | --- | --- | --- | --- |
|  |  | **1** | **2** | **3** | **4** |
| A | pH | 3 | 5 | 7 | 9 |
| B | Temperature [°C] | 20 | 25 | 30 | 35 |
| C | Concentration of NaCl [%] | 0 | 5 | 10 | 15 |
| D | Medium volume [%] | 15 | 20 | 30 | 40 |

**Table S6** Taguchi L16 Orthogonal Array. Letters correspond to factors, while numbers refer to levels.

| **RUN** | **A** | **B** | **C** | **D** |
| --- | --- | --- | --- | --- |
| **1** | 1 | 1 | 1 | 1 |
| **2** | 1 | 2 | 2 | 2 |
| **3** | 1 | 3 | 3 | 3 |
| **4** | 1 | 4 | 4 | 4 |
| **5** | 2 | 1 | 2 | 3 |
| **6** | 2 | 2 | 1 | 4 |
| **7** | 2 | 3 | 4 | 1 |
| **8** | 2 | 4 | 3 | 2 |
| **9** | 3 | 1 | 3 | 4 |
| **10** | 3 | 2 | 4 | 3 |
| **11** | 3 | 3 | 1 | 2 |
| **12** | 3 | 4 | 2 | 1 |
| **13** | 4 | 1 | 4 | 2 |
| **14** | 4 | 2 | 3 | 1 |
| **15** | 4 | 3 | 2 | 4 |
| **16** | 4 | 4 | 1 | 3 |


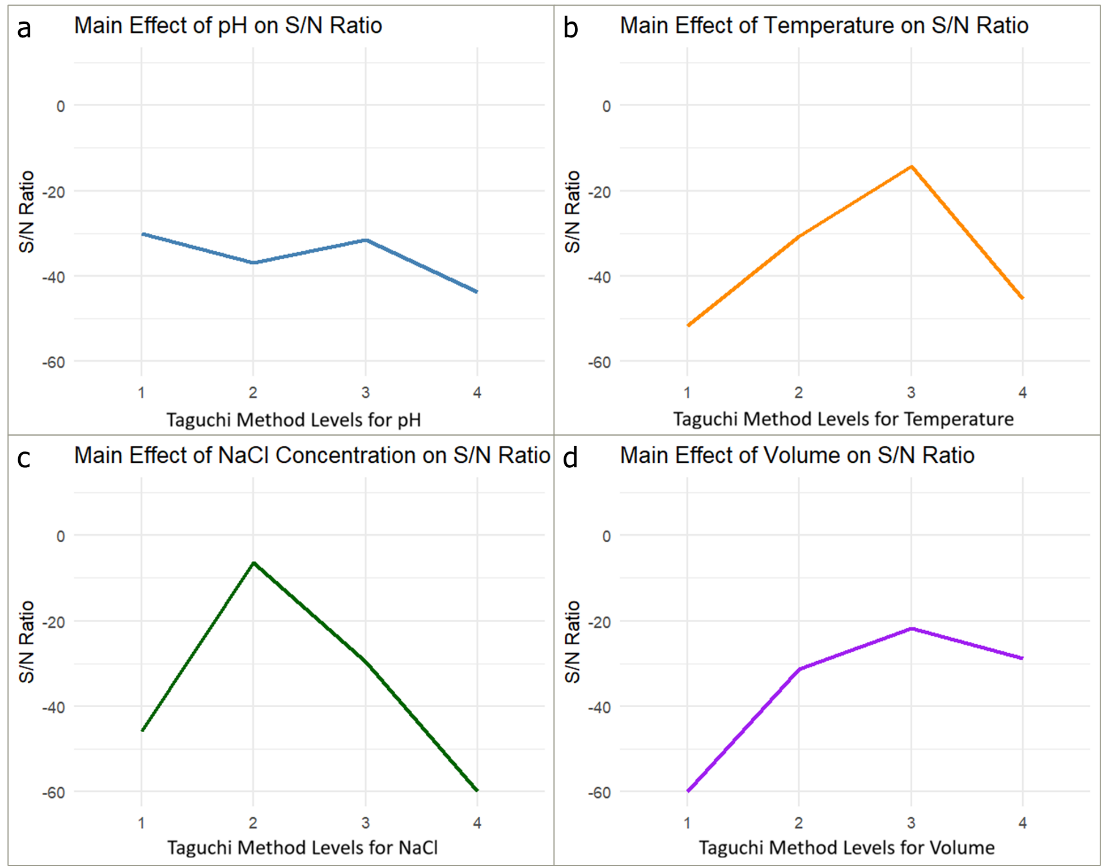


**Figure S8** Main effect plots for S/N ratios of Taguchi experiment for a) pH,
b) temperature, c) sodium chloride concentration, and d) volume. Levels were described in Table S1.

**References:**

1. Jain C, Rodriguez-R LM, Phillippy AM, Konstantinidis KT, Aluru S. 2018. High throughput ANI analysis of 90K prokaryotic genomes reveals clear species boundaries. Nat Commun 9:1–8.

2. Ondov BD, Treangen TJ, Melsted P, Mallonee AB, Bergman NH, Koren S, Phillippy AM. 2016. Mash: Fast genome and metagenome distance estimation using MinHash. Genome Biol 17:1–14.
